# Continuous bioactivity-dependent evolution of an antibiotic biosynthetic pathway

**DOI:** 10.1038/s41467-020-18018-2

**Published:** 2020-08-21

**Authors:** Chad W. Johnston, Ahmed H. Badran, James J. Collins

**Affiliations:** 1 Institute for Medical Engineering and Science, Massachusetts Institute of Technology, 77 Massachusetts Ave, Cambridge, MA 02139 USA; 2 Broad Institute of MIT and Harvard, 415 Main St, Cambridge, MA 02142 USA; 3 Wyss Institute for Biologically Inspired Engineering, Harvard University, 3 Blackfan Circle, Boston, MA 02115 USA; 4 Department of Biological Engineering, Massachusetts Institute of Technology, 77 Massachusetts Ave, Cambridge, MA 02139 USA; 5 Synthetic Biology Center, Massachusetts Institute of Technology, 77 Massachusetts Ave, Cambridge, MA 02139 USA; 6 Harvard-MIT Program in Health Sciences and Technology, 77 Massachusetts Ave, Cambridge, MA 02139 USA

**Keywords:** Metabolic engineering, Natural product synthesis, Synthetic biology

## Abstract

Antibiotic biosynthetic gene clusters (BGCs) produce bioactive metabolites that impart a fitness advantage to their producer, providing a mechanism for natural selection. This selection drives antibiotic evolution and adapts BGCs for expression in different organisms, potentially providing clues to improve heterologous expression of antibiotics. Here, we use phage-assisted continuous evolution (PACE) to achieve bioactivity-dependent adaptation of the BGC for the antibiotic bicyclomycin (BCM), facilitating improved production in a heterologous host. This proof-of-principle study demonstrates that features of natural bioactivity-dependent evolution can be engineered to access unforeseen routes of improving metabolic pathways and product yields.

## Abstract (web summary)

Biosynthetic gene clusters (BGCs) make small molecules with fitness-enhancing activities that drive BGC evolution. Here, the authors show that synthetic biology can leverage bioactivity to achieve continuous evolution of an antibiotic BGC in the lab and improve antibiotic production in a new host.

## Introduction

Antibiotic BGCs are found in nearly all sequenced bacteria, where their genes, proteins, and chemical products continuously undergo natural selection, evolving their fitness-enhancing bioactivities^1^. This bioactivity-dependent selection pressure can favor the retention and adaptation of BGCs acquired through horizontal gene transfer (HGT) from related organisms^2^, phylogenetically divergent bacteria^3^, or even other kingdoms of life^4^. The sampling of untargeted mutations during natural BGC evolution contrasts with genetic refactoring methods used in synthetic biology that rely on regulatory element exchange and codon optimization to adapt BGCs and improve metabolite yield^5^. Bioactivity-dependent directed evolution presents a complementary approach to improve metabolite production.

In vivo directed evolution requires strategies for mutation, selection, and amplification of genes of interest (GOIs). In nature, these processes are coupled and form the basis for Darwinian evolution through natural selection. Continuous directed evolution approaches have found utility in mirroring these coupled cycles of diversification and selection^6–8^, with phage-assisted continuous evolution (PACE)^9^ being one of the most well-studied methods^10–12^. In PACE, engineered bacteriophage carrying an evolving GOI persist in mutagenic lagoons where their infectivity is dependent upon the bioactivity of the GOI product, enabling continuous cycles of diversified replication and selection. Here, we extend these capabilities to the continuous directed evolution of an antibiotic BGC and demonstrate PACE’s ability to drive mechanism-agnostic optimization of a biosynthetic pathway.

To investigate whether PACE could improve antibiotic production, we haven chose to work with the antibacterial agent bicyclomycin (BCM)^3,13,14^, which is a unique inhibitor of the bacterial transcription termination factor Rho^15^. BCM is a modified 2,5-diketopiperazine, biosynthesized by a seven-enzyme pathway that is initiated by a tRNA-dependent cyclodipeptide synthase to form a cyclo(L-Ile-L-Leu) core that is subsequently oxidized by five non-heme iron-dependent monooxygenases and one cytochrome P450 monooxygenase^14^. First discovered in *Streptomyces*^16^, BCM BGCs have been identified in Gram-negative bacteria^3,17^, where they appear to have been acquired and adapted following HGT^3^. In this work, we demonstrate that this same bioactivity-dependent selection pressure can be used to adapt this BGC and improve the yield of its antibiotic product in a heterologous host.

## Results

### Development of a genetic circuit to detect BCM bioactivity

**Fig. 1:**
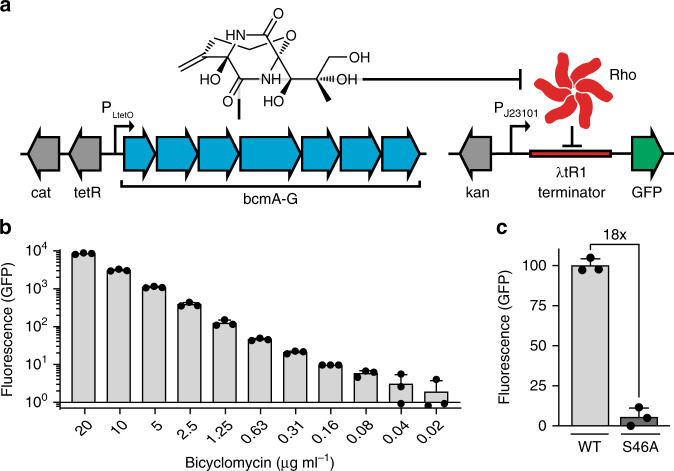
A genetically encoded sensor for BCM bioactivity. **a** Schematic representation of plasmids for BCM production and BCM-dependent GFP expression, featuring a constitutive promoter interrupted by the Rho-dependent λtR1 terminator. BCM biosynthesis from a plasmid carrying an anhydrotetracycline (aTC) inducible promoter results in GFP production by alleviating Rho-dependent transcriptional termination of GFP on reporter plasmid pREP.1. **b** GFP reporter pREP.1 response across a wide range of BCM concentrations. **c** GFP production upon induction of the *P. aeruginosa* wild-type (WT) or inactivated (S46A) BCM operon. All data shown as mean ± s.d.; *n* = 3 biological replicates. Source data underlying **b**, **c** are available as a Source Data file.

Positive selection during PACE requires linking gene expression to an evolving bioactivity. We developed a synthetic gene circuit wherein BCM production would trigger expression of a reporter protein in a dose-dependent manner. Our sensor features a constitutive promoter driving GFP transcription, interrupted by a Rho-dependent λtR1 terminator. BCM-dependent Rho inhibition derepresses reporter transcription, resulting in GFP production (Fig. 1a, right). Addition of a BCM standard to *Escherichia coli* cells carrying this reporter revealed a robust correlation between BCM concentration and GFP fluorescence, with nearly 1000-fold dynamic range and sensitivity to antibiotic levels far below the MIC of ~25 μg mL^−1^ (Fig. 1b). Reporter sensitivity and signal-to-noise could be altered by changing promoter strength and plasmid or terminator copy number (Supplementary Figs. 1 and 2).

To validate that this circuit design could facilitate bioactivity-dependent positive selection in vivo, we tested it in the presence of an inducible BCM operon taken from *Pseudomonas aeruginosa*^13^ (Fig. 1a). Comparison of *E. coli* carrying the sensitive BCM reporter plasmid pREP.1 and either a wild-type or catalytically inactivated^18^ (BcmA S46A) BCM expression plasmid (EP; pBCM.1) revealed 18-fold higher fluorescence in cells carrying the functional operon (Fig. 1c). Consistent with prior enzymatic characterization^3,13,14^, BCM production was optimal at ≤30 °C and ablated at temperatures above 33 °C (Supplementary Fig. 3). To demonstrate bioactivity-dependent selection and improve our signal-to-noise before PACE, mutations were introduced into pBCM.1 by error-prone PCR and passage through mutagenic *E. coli*, and resultant clones were induced in the presence of the GFP reporter and sorted by fluorescence-activated cell sorting (FACS). This approach identified mutagenized operons with a fivefold improvement in reporter signal relative to the wild-type operon (Supplementary Fig. 4), providing an optimized starting point for PACE.

### Bioactivity-dependent PACE of BCM biosynthesis

**Fig. 2:**
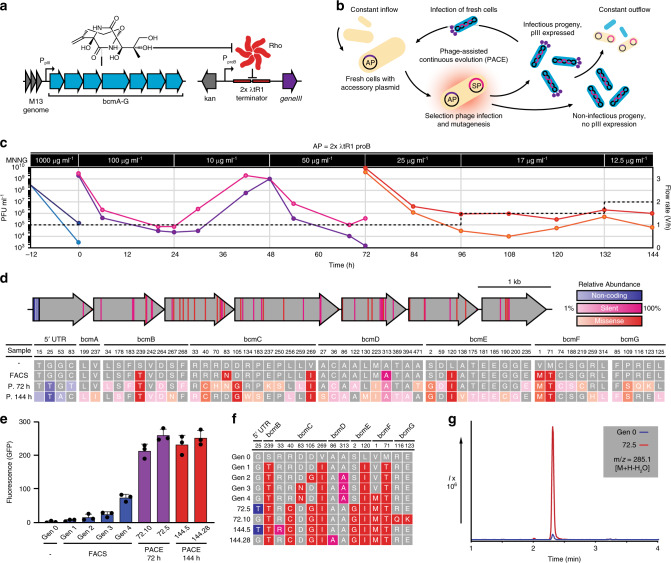
Bioactivity-dependent continuous evolution of the *P. aeruginosa* BCM BGC. **a** Accessory plasmid and genetic circuit design for bioactivity-dependent expression of *geneIII*. **b** Schematic representation of PACE. *E. coli* cells carrying an accessory plasmid (AP; purple) encoding *geneIII* are provided continuously from a chemostat to fixed-volume lagoons inoculated with selection phage (SP; blue). Only functional SPs can propagate and persist within the lagoon under continuous dilution. Lagoons are supplemented with a chemical mutagen to facilitate mutagenesis at 30 °C. **c** SP_BCM_ titers (plaque forming units; PFU) over 144 h of PACE, reporting values for duplicate lagoons. Concentrations of the mutagen 1-methyl-3-nitro-1-nitrosoguanidine (MNNG) are indicated for each segment, along with lagoon flow rates (dashed lines). **d** Summary of mutations accumulated during BCM operon evolution. Below, relative abundance of each mutation is depicted. **e** Activity of wild-type and evolved BCM operons against the stringent reporter pREP.2 (Supplementary Fig. 1). Shown as mean ± s.d.; *n* = 3 biological replicates. **f** Genotypes of tested BCM variants. A legend for color coding can be found in **d**. **g** LC/MS extracted ion chromatograms of the dominant bicyclomycin parent ion (an in-source decay water loss, m/z = 285.1 [M + H–H_2_O]) from culture supernatants of the original BCM operon (Gen 0) and a PACE-evolved variant (72.5). Source data underlying **c**, **e** are available as a Source Data file.

The stringent BCM reporter plasmid pREP.2 (Supplementary Fig. 1) was modified to create an accessory plasmid (AP) for PACE, such that increasing concentrations of BCM result in increasing expression of the phage infectivity protein pIII (the product of *geneIII*; Fig. 2a). Selection phage (SP) bearing our FACS-optimized BCM operon (SP_BCM_) efficiently produced progeny from infected *E. coli* S2060 cells carrying our AP, while those bearing the BcmA S46A mutant did not (Supplementary Fig. 5). To accommodate the observed temperature sensitivity, PACE campaigns were carried out at 30 °C using the chemical mutagen 1-methyl-3-nitro-1-nitrosoguanidine (MNNG) for mutagenesis (Fig. 2b). To maximize the chances of discovering highly functional BCM operons, we first subjected SP_BCM_ to a high concentration of MNNG (1 mg mL^−1^) to diversify our starting population, and observed a decline in phage titers consistent with a high mutagenic burden (Fig. 2c). This diversified population was subjected to 144 h of PACE—equivalent to >800 cycles of phage replication—wherein mutagenesis rate and selection stringency were modified by changes to MNNG concentration and lagoon flow rate, respectively. Phage samples were collected at 72 and 144 h, and evolved operons were assessed using GFP reporter assays (Supplementary Figs. 6 and 7).

Sequence analysis of our evolved operons revealed a diversified pool of active BGCs (Fig. 2d), including a series of clones marked by a BcmC R40C mutation that demonstrated over tenfold higher reporter signal relative to our wild-type operon (Fig. 2e, f). This improved yield was further supported by liquid chromatography–mass spectrometry (LC/MS) analysis of BCM in culture extracts (Fig. 2g). From 72 sequenced operons, we observed 51 new mutations, including 30 silent or noncoding mutations and 21 missense mutations, many of which were also observed in sequenced BCM operons in other organisms (Supplementary Data 1, Supplementary Data 2). To assess how these mutations were impacting BCM production, individual mutant genes were cloned into a wild-type operon and induced in the presence of our reporter (Supplementary Fig. 8), revealing that most sequentially acquired mutations worked additively to improve production. To determine whether improved protein solubility was responsible for the observed increase in BCM production, we used wild-type and mutant versions of our enzymes in a split T7 RNA polymerase complementation assay^19^ (Supplementary Fig. 9). Final mutant versions of our Bcm enzymes generally did not possess greatly improved solubility—aside from BcmE L120I S2G—but several of the intermediate mutant enzymes did, which appears to have facilitated access to new sets of mutations that improved BCM production at the cost of soluble expression. LC/MS analysis of BCM metabolites in cultures expressing the wild-type or high-yield evolved operon revealed considerable changes in the relative abundance of key intermediates (Supplementary Fig. 10), suggesting that adaptations in enzyme functionality compensate for the metabolic environment in *E. coli*. When these same operons are expressed in *P. fluorescens* SBW25—which is closely related to the organism from which this operon was sourced—we did not observe improved BCM production (Supplementary Fig. 11), indicating again that PACE adapted this operon to improve BCM production in our heterologous host. While quantitative LC/MS analysis revealed a 20-fold increase in yield between our evolved and wild-type operons in *E. coli* (0.6 and 0.03 μg mL^−1^, respectively), this effect was reversed in the previously described *P. fluorescens* BCM expression system (1.4 and 4.8 μg mL^−1^ respectively), where wild type yields closely matched those from the associated expression plasmid^3^ (6 μg mL^−1^, Supplementary Fig. 12).

### Improving population fitness by tuning selection difficulty

**Fig. 3:**
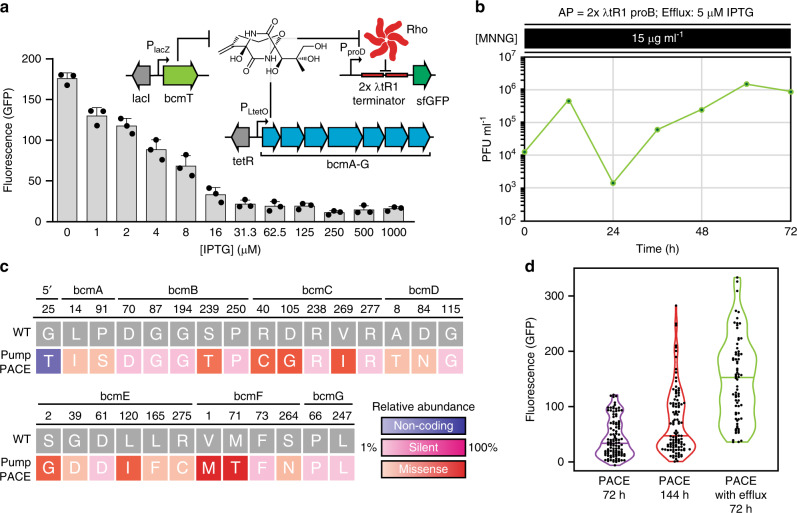
BCM efflux during PACE yields a more productive population of BGCs. **a** Fluorescence (GFP) signal from BCM reporter plasmid pREP.2 in *E. coli* cells carrying the IPTG-inducible BCM efflux pump expression plasmid pPUMP and evolved BCM production plasmid pBCM.1 (recovered PACE clone 72.5). Results are absorbance normalized signal over uninduced controls, shown as mean ± s.d.; *n* = 3 biological replicates. **b** SP_BCM_ titers (plaque forming units, PFU) over an additional 72 h of PACE, using S2060 *E. coli* carrying accessory plasmid pAP.1 and the IPTG-inducible efflux pump expression plasmid pPUMP (induced with 5 μM IPTG), along with 15 μg mL^−1^ MNNG for mutagenesis. Lagoon flow rate was maintained at 1 vol h^−1^. **c** Summary and relative abundance of mutations observed in BCM operons following PACE with active BCM efflux (Pump PACE) from 24 sequenced operons (Supplementary Fig. 14). **d**. Summary of assayed BCM operon populations recovered from PACE (Supplementary Figs. 6, 7, and 14). The median value is marked with a line in each violin plot. Source data underlying **a**, **b**, **d** are available as a Source Data file.

PACE is unique among continuous evolution methods in that the replication of evolving material is uncoupled from host replication for genetic selections. This allows PACE to evolve potentially toxic biomolecules or phenotypes that could be harmful to *E. coli*, as naive cells are provided continuously, and their replication does not serve as the basis for selection. However, as our selection makes use of endogenous machinery, our capacity for selection is theoretically limited by the potency of the antibiotic and the abundance of its target. In order to dynamically modulate selection capacity and stringency during PACE—and limit potential toxicity downstream during production in *E. coli*—we tested a chemically inducible BCM efflux pump expression plasmid (*bcmT*; pPUMP). In cells carrying pPUMP, BcmT provided resistance to BCM (Supplementary Fig. 13a), and increasing expression resulted in lower reporter signal in response to BCM provided exogenously (Supplementary Fig. 13b) or produced inside the cell (Fig. 3a). To assess how active efflux would impact bioactivity-dependent evolution, we ran a 72 h PACE experiment, starting from our evolved phage pool. Reflecting the increased selection stringency imposed by active efflux of BCM during PACE, we observed lower overall phage counts, along with retention and evolution of only our most productive operons from the evolved phage pool (Fig. 3, Supplementary Fig. 14). Plotting the populations of all recovered and assayed BGCs throughout our PACE experiments demonstrates that increasingly difficult selections can eliminate unproductive clones and more effectively drive a population-level shift toward increasingly active BGCs (Fig. 3d). As efflux pumps are associated with nearly all antibiotic BGCs, they could provide a universal mechanism to dynamically control selection difficulty during BGC evolution.

## Discussion

Bioactivity-dependent evolution of antibiotic biosynthesis in PACE emulates natural selection by continuously introducing and sampling genetic diversity to promote fitness in the population. As each gene in a biosynthetic pathway contributes to producing a metabolite with a fitness-enhancing bioactivity, evolving the entire gene cluster simultaneously can provide access to unexpected mechanisms of improving yields that would not be identified by focusing on individual biosynthetic components^20^. Importantly, the mutations and adaptations accessed through bioactivity-dependent BGC evolution should be complementary to sequence optimizations featured in traditional genetic refactoring approaches^5^. Because PACE decouples the replication of the evolving GOI from the replication of hosts for selection, it can be used to evolve potentially toxic phenotypes—such as antibiotic production—that would limit other, less segregated systems, where selection depends on host viability and reproduction^6–8^.

Throughout this study, we observed that increasing the difficulty of a given genetic selection improved the overall activity of the evolving population at the cost of sequence diversity. As we had previously seen that epistatic mutations could provide access to improved properties—including protein solubility—it should be noted that strong selection pressure may not necessarily drive evolution toward a global fitness maxima. Conveniently, antibiotic BGCs possess conserved features that can be used to dynamically adjust selection pressure during continuous bioactivity-dependent evolution. Antibiotic resistance genes are endemic to BGCs^21^, where their expression is typically driven by a colocalized transcriptional regulator that is responsive to an associated antibiotic’s chemical structure^22^ or to its specific mechanism of action^23^. Resistance enzymes that inactivate antibiotics or remove them from the cell can also be induced during directed evolution, such that their expression can be titrated to gradually adjust selection stringency. As we demonstrated with the induced expression of the BCM efflux pump *bcmT*, this additional pressure can be used to eliminate unproductive clones, limit potential cross-talk caused by antibiotic transmission between cells, and mitigate toxicity. While it had initially seemed likely that intracellular antibiotic accumulation during evolution was limiting our access to more productive sequences, analysis of clones recovered from PACE with active BCM efflux demonstrated that, in this case, toxicity was a minor limitation. Still, given that bioactivity-dependent continuous evolution relies on gene expression being linked to inhibition of an intracellular target, the upper limit on attainable antibiotic yields would be bound by the dynamic range of the genetic response to bioactivity, target affinity, antibiotic-associated toxicity, and the abundance and rates of antagonizing resistance enzymes.

Continuous directed evolution methods rely on genetic selections that are tailored to the current or desired function of the evolving GOI. For bioactivity-dependent continuous evolution, these selections instead rely on the activity of the small molecule produced by an evolving biosynthetic pathway. PACE mandates that the function of the GOI be linked to expression of the minor-coat protein pIII, a criterion that has been addressed with unique selection circuits in order to facilitate the evolution of nucleotide-binding^24^ or nucleotide-modifying enzymes^25^, transcription^9,11^ and translation^10^ machinery, or protein–protein interactions^12^. In this proof-of-principle study, we demonstrated that the biosynthetic pathway for BCM could be evolved with PACE by leveraging BCM’s unique mechanism of action. Although other antibiotics do not share the same this antibacterial mechanism, the selection tactic employed in this approach—linking antibiotic-mediated target inhibition to gene expression—could be applied to many other antibiotic classes. Generalization of this platform thus relies on the development of new gene circuits that can select for inhibition of different biological targets^21,26^, with the most readily accessible likely being those directly related to gene expression and the central dogma. Orthogonal systems^27^ for transcription and translation could be used to create gene circuits where enzyme inhibition does not impact cellular fitness, facilitating the evolution of antibiotics that affect RNA polymerases^28^, ribosomes^29^, and tRNA synthetases^30^, among others. Abstraction from biological function could be taken further by employing the established two-hybrid PACE system^12^ to evolve BGCs for antibiotics that facilitate^31^ or disrupt protein–protein interactions^32^. Alternatively, transcriptional regulators of resistance genes can be known to detect the damage caused by specific antibiotics, and leveraging these proteins could provide a bioactivity-dependent means of evolving valuable drugs like carbapenems^23^. Ultimately, directed evolution schemes are primarily limited by the creativity and biological understanding behind the selection structure. As we demonstrated in this study, so long as gene expression can be tied to the activity of an evolving molecule or BGC, PACE could be a viable strategy to emulate natural bioactivity-dependent evolution and drive the improvement and adaptation of antibiotic biosynthetic pathways.

## Methods

### General methods

DNA cloning, BCM expression studies, and GFP assays were performed in NEB Turbo cells (New England Biolabs) grown in 2× LB media (BD Biosciences). All discrete infection assays, plaque assays, and PACE experiments were performed in *E. coli* S2060 cells^11^ grown in Davis Rich Media (DRM). For BCM production and LC/MS analysis, *E. coli* cells were grown in DRM, while *P. fluorescens* SBW25 cells were grown in BCM production media (BCMM)^3^. Plasmids and SPs were assembled with USER cloning, and amplified using Phusion U Hot Start DNA polymerase (Thermo Fisher Scientific). Antibiotics were used at the following concentrations: carbenicillin, 50 μg mL^−1^; tetracycline, 10 μg mL^−1^; chloramphenicol, 50 μg mL^−1^; kanamycin, 25 μg mL^−1^. DNA for Rho-dependent terminators and for assembling the *P. aeruginosa* BCM pathway was synthesized by Integrated DNA Technologies. A list of all primers used in this study is available in Supplementary Data 3, and a list of all plasmids used in this study is available in Supplementary Table 1.

### Preparation and transformation of competent cells

For creating competent cells of NEB Turbo or S2060 *E. coli*, overnight cultures were inoculated into 50 mL 2× LB media (BD Biosciences) and grown in an incubator set at 37 °C and 290 rpm to mid-log-phase (absorbance at 600 nm = 0.5). Cells were harvested by centrifugation at 4000 *g* at 4 °C for 10 min. Cell pellets were resuspended in 1.25 mL of 2× TSS solution (LB media containing 10% PEG 3350, 5% v/v DMSO, and 20 mM MgCl_2_). Aliquots were frozen at −80 °C for later use.

For transformations, 50 μL aliquots of cells suspended in TSS were thawed on ice and 7.5 μL of KCM solution (100 mM KCl, 30 mM CaCl_2_, and 50 mM MgCl_2_) was added, along with the plasmid DNA or USER assembly reaction to be transformed. Cell–DNA mixtures were left on ice for 15 min before heat shock in a 42 °C water bath for 45 s. Transformations were cooled on ice for 1 min before the addition of 750 μL of 2× LB and transfer to a shaking 37 °C incubator for recovery over 45 min. Following recovery, cells were centrifuged at 4000 *g*, resuspended in a small volume of residual media, and plated on 2× LB plates containing appropriate antibiotics, growing out overnight at 37 °C.

To create electro-competent *P. fluorescens* SBW25 cells, single colonies were inoculated into 4 mL of 2× LB media (BD Biosciences) in a 15 mL polypropylene culture tube and grown in an incubator set at 28 °C and 300 rpm overnight. On the following day, cells were harvested by centrifugation at 4000 *g* at 4 °C for 10 min. Cells were washed twice with ice-cold sterile 10% glycerol before being resuspended in 200 μL 10% glycerol. Aliquots of 50 mL cell suspension mixed with DNA were electroporated with 2500 V, resuspended in 1 mL 2× LB, and recovered at 28 °C with shaking for 1 h before being plated on LB agar plates with appropriate antibiotics at 28 °C.

### Taq mutagenesis

For error-prone PCR mutagenesis, the BCM operon was amplified with Taq DNA polymerase (New England Biolabs) in 96 × 50 μL reactions. PCR amplicons were isolated with a QIAquick PCR purification kit (Qiagen), treated with DpnI (New England Biolabs) to remove residual template DNA, and then gel extracted. This purified pool of mutated BCM operons was amplified again in 96 × 50 μL PCR reactions, using Phusion U polymerase and primers containing deoxyuridine to install USER-compatible termini for assembly. Amplicons were PCR purified, treated with DpnI, and gel extracted before USER assembly with an aTC-inducible plasmid backbone. 72 × 10 μL USER assembly reactions were pooled and transformed into fresh TSS-KCM competent NEB Turbo *E. coli* cells carrying our superfolder GFP BCM reporter plasmid (equivalent to 100 mL of culture with absorbance at 600 nm = 0.5). Following a 15 min incubation on ice, cells were heat shocked in a 42 °C water bath for 45 s. Transformations were cooled on ice for 1 min before being diluted into 500 mL of 2× LB, shaking in a 37 °C incubator for 45 min to recover. Following this recovery, antibiotics were added for selection of reporter and BCM plasmids. Following overnight growth, this pooled transformation was diluted 1:100 into 100 mL of fresh 2× LB with antibiotics and 100 ng mL^−1^ aTC to induce BCM production, and left growing for 12–16 h at 30 °C and 290 rpm. After overnight outgrowth and induction, cells were diluted 1:1000 into 1× PBS and sorted using a BD FACSAriaII (BD Biosciences), gating forward and side scatter to enrich for single cells, then sorting based on superfolder GFP fluorescence intensity. Sorted cells suspended in PBS were plated on 2× LB agar with appropriate antibiotics and allowed to grow for 12–16 h at 37 °C. Three hundred eighty-four individual colonies of sorted cells were picked and resuspended into individual wells of a deep 96-well plate containing 2× LB with antibiotics. Plates were grown at 37 °C and 900 rpm until cells reached early–mid log phase (absorbance at 600 nm = 0.2), after which individual wells were diluted into fresh media in four wells in a new plate, including duplicates with and without aTC. New plates were grown for 12–16 h at 30 °C and 900 rpm before cell density (absorbance at 600 nm) and superfolder GFP fluorescence from the reporter was measured on a SpectraMax plate reader (Molecular Devices) to assess BCM production from mutated plasmids recovered following Taq mutagenesis.

### XL-1 Red evolution

Fifty microliters aliquots of XL-1 Red *E. coli* cells (Agilent) were thawed on ice for 15 min before 1 μL of a purified plasmid carrying an inducible copy of the BCM operon was added. Following 15 min incubation on ice, cells were heat shocked in a 42 °C water bath for 45 s. Transformations were cooled on ice for 1 min before the addition of 750 μL of 2× LB and transfer to a shaking 37 °C incubator for recovery over 45 min. Following this recovery, transformations were plated on 2× LB agar containing 50 μg mL^−1^ chloramphenicol and 10 μg mL^−1^ ketoconazole, incubating overnight at 37 °C. Colonies of transformants were washed off the plates and resuspended in 2× LB containing matching antibiotics, diluted into a 96-well plate, and grown overnight at 37 °C. Every 24 h for 3 days, the plate was diluted 1:100 into new media and the remaining culture was used to isolate mutated BCM plasmids with a QIAprep Spin Miniprep kit (Qiagen). Following serial passaging and DNA prep, a pooled, mutated plasmid sample was transformed into competent NEB Turbo cells carrying a BCM-sensitive superfolder GFP reporter plasmid, and was allowed to recover in 100 mL of 2× LB for 45 min before the addition of antibiotics, followed by outgrowth for 12–16 h at 37 °C and 290 rpm. Following overnight outgrowth, transformed NEB Turbo cells carrying our BCM-sensitive reporter plasmid and our mutated BCM biosynthesis plasmid were diluted 1:100 into 100 mL fresh 2× LB media with antibiotics and 100 ng mL^−1^ aTC to induce BCM production, and left growing for 12–16 h at 30 °C and 290 rpm After overnight outgrowth and induction, cells were diluted 1:1000 into 1× PBS and sorted using a BD FACSAriaII (BD Biosciences), gating forward and side scatter to enrich for single cells, then sorting based on superfolder GFP fluorescence intensity. Sorted cells suspended in PBS were plated on 2× LB agar with appropriate antibiotics and allowed to grow for 12–16 h at 37 °C. Three hundred eighty-four individual colonies of sorted cells were picked and resuspended into individual wells of a deep 96-well plate containing 2× LB with antibiotics. Plates were grown at 37 °C and 900 rpm until cells reached early–mid log phase (absorbance at 600 nm = 0.2), after which individual wells were diluted into fresh media in four wells in a new plate, including duplicates with and without aTC. New plates were grown for 12–16 h at 30 °C and 900 rpm before cell density (absorbance at 600 nm) and superfolder GFP fluorescence from the reporter were measured on a SpectraMax plate reader (Molecular Devices) to assess BCM production from mutated plasmids recovered from XL-1 Red transformants.

### GFP reporter assays

Single colonies of NEB Turbo *E. coli* cells carrying a reporter plasmid and potentially also a plasmid containing an inducible BCM operon were used to create glycerol stocks. For assays, 2× LB containing appropriate antibiotics was inoculated with an appropriate stock, sampled with a sterile wooden stick. Cultures were grown overnight at 37 °C and 250 rpm to recover. On the following day, fresh media was inoculated 1:1000 with the overnight cultures grown in similar conditions to absorbance at 600 nm = 0.6–0.8 before being diluted 1:100 into 300 μL of fresh media in a deep 96-well plate for assays. For reporter dose-response assays, 2× LB with antibiotics was serially diluted with BCM (dissolved in methanol; Santa Cruz Biotechnology) and warmed to 30 °C prior to the addition of cells. For assays involving the induction of the BCM operon, 2× LB with antibiotics and with or without 100 ng mL^−1^ aTC was added to a deep 96-well plate and warmed to the appropriate temperature (30 °C in all cases beyond temperature dependence assays) prior to the addition of cells. Following inoculation, the assay plate was sealed with a breathable membrane and shaken at 900 rpm for 12 h. After incubation with shaking, assay plates were sampled, measuring fluorescence, and absorbance of the cultures on a SpectraMax plate reader (Molecular Devices). For assays involving the BCM efflux pump (*bcmT*), IPTG for induction of pump expression was added simultaneously with aTC and/or BCM.

### Luciferase assay

S2060 cells were transformed with the pTW004(s) and pTW006 plasmids of interest, carrying the T7 RNA polymerase N-terminal fusions of Bcm proteins, and the T7 C-terminal fragment and luciferase reporter, respectively^19^. Single colonies were grown overnight in DRM media supplemented with maintenance antibiotics, and were then diluted 1000-fold into DRM media with maintenance antibiotics in a deep 96-well plate (VWR). The plate was sealed with a porous sealing film and grown at 37 °C with shaking at 900 rpm for 4 h, whereupon the culture reached OD600 ~0.4–0.6. Cells were induced with 100 ng mL^−1^ aTC and 5 µM arabinose before incubation for an additional 1 h at 37 °C with shaking at 900 rpm, before 150 µL of cells were transferred to a 96-well black-walled clear-bottomed plate to measure absorbance at 600 nm and luminescence using a Tecan Spark microplate reader. OD600-normalized luminescence values were obtained by dividing raw luminescence by background-subtracted 600 nm absorbance. The background value was set to the 600 nm absorbance of wells containing DRM only.

### Liquid chromatography and mass spectrometry

Mass spectrometry was performed using a Thermo QExactive mass spectrometer (ThermoFisher Scientific, USA) with a HESI II probe, operated in positive mode. Liquid chromatography was performed with a coupled Thermo Dionex UltiMate 3000 HPLC system (ThermoFisher Scientific, USA), using a Luna Omega 1.6 μm Polar C18 column (50 × 2.1 mm, Phenomenex) maintained at 25 °C. Mobile phases were water with 0.1% formic acid or methanol with 0.1% formic acid. For separations, organic solvent was increased from 0 to 35% over 6 min, followed by a wash at 100% starting at 6 min 6 s. BCM fragmentation and retention time were confirmed via analytical standard (Santa Cruz Biotechnology) prior to analysis. High-resolution mass spectrometry data were collected with full scan mode and with parallel reaction monitoring (PRM) for quantification. PRM targeting of BCM was accomplished by filtering for the associated ion (m/z = 285.1 [M + H–H_2_O]) and subsequent diagnostic MS/MS detection of diagnostic fragment ions. Intensities and peak areas of BCM were measured with MZmine2 software^33^ and concentrations were calculated by comparison with a standard curve.

For analysis of BCM production in *E. coli*, NEB Turbo cells were transformed with expression plasmid pBCM.1 or expression and efflux plasmid pBCM.2 bearing either the original BCM sequence or an evolved gene cluster recovered from PACE. Single colonies were grown overnight at 37 °C and 300 rpm in 4 mL DRM with appropriate antibiotics. Following overnight growth, a second set of 4 mL cultures was inoculated 1:100 and grown to absorbance at 600 nm = 0.6–0.8, then diluted 1:100 into 4 mL DRM with antibiotics and 100 ng mL^−1^ aTC and grown at 28 °C and 300 rpm for 16 h. Cultures were harvested by centrifugation at 3200 *g* for 15 min at 4 °C using an Eppendorf 5804R centrifuge. Supernatants were sterilized by syringe filtration (0.2-μm filter) and cell pellets were resuspended in methanol, followed by a second centrifugation.

For analysis of BCM production in *P. fluorescens*, we followed the protocol outlined by Vior et al.^3^. Briefly, cells were transformed with *Pseudomonas* protein expression vector pJH, carrying a wild-type or PACE-evolved BCM operon, along with the BCM efflux pump *bcmT*. Single colonies of the resulting transformations were used to inoculate 4 mL cultures of BCMM in 15 mL polypropylene culture tubes, growing overnight at 28 °C and 300 rpm Following overnight growth, a 400 μL aliquot was diluted into 10 mL fresh BCMM in a 50 mL Falcon tube and covered with a foam bung. Production cultures were grown for 16 h at 28 °C and 300 rpm Cultures were harvested by centrifugation at 3200 *g* for 15 min at 4 °C using an Eppendorf 5804R centrifuge. Supernatants were sterilized by syringe filtration (0.2-μm filter).

### Plaque assays

Single colonies of S2060 *E. coli* cells carrying the activity-independent persmissive plasmid pJC175e^11^ were grown overnight in DRM media supplemented with antibiotics, before being diluted 1:1000 into fresh DRM media with antibiotics and grown at 37 °C and 250 rpm to absorbance at 600 nm = 0.6–0.8 before use. Phage samples were serially diluted 1:100 four times in DRM media. 100 μL of cells were added to 10 μL of each phage dilution and allowed to sit for ~1 min before addition of 1 mL of warm liquid top agar (2× YT media + 0.4% agar) supplemented with 2% Bluo-gal (dissolved in DMF; Sigma) to a final concentration of 40 μg mL^−1^ was added and mixed by pipetting up and down once. The resuspended cells and phage dilution were then pipetted onto a quartered Petri dish already containing 2 mL of solidified bottom agar in each quadrant (2× YT media + 1.5% agar, no antibiotics). Plates were incubated at 37 °C overnight after the top agar was observed to have solidified.

### Phage propagation assays

Single colonies of S2060 cells carrying an AP of interest were prepared similarly to those used in activity-independent plaque assays. Following dilution into fresh media and growth at 37 °C and 250 rpm to absorbance at 600 nm = 0.6–0.8, 4 mL cultures of cells were divided into two new cultures and prepared SP were added to a final titer of 1 × 10^5^ pfu mL^−1^. Cultures were moved to a 30 °C shaker set to 250 rpm and left for phage propagation. Phage counts were assessed after 2, 4, and 6 h, collecting supernatants containing phage by transferring 100 μL of culture to a 1.7 mL Eppendorf tube, centrifuging at 2000 *g* for 5 min. Supernatants were filtered using sterile polyethersulfone 0.2-μm centrifuge filters (mdi Membrane Technologies), centrifuging again at 2000 *g* for 5 min. Samples were stored at 4 °C for up to 1 week before use.

### Phage-assisted continuous evolution

The apparatus for PACE—including tubing, pumps, chemostats, and lagoons—was used as previously described^9,11,12^ unless noted otherwise. S2060 cells were transformed with the AP pAP.1 and plated on 2× LB agar containing appropriate antibiotics, incubating overnight at 37 °C. A single colony of S2060 with the transformed AP was grown in 4 mL DRM with antibiotics at 37 °C and 250 rpm until absorbance at 600 nm = 0.6–0.8, when it was added to a 100 mL chemostat containing fresh media. The chemostat culture was grown until absorbance at 600 nm = 0.6–1.0, after which pumps were started to supply fresh media at a rate of 1–1.5 vol h^−1^ to maintain cell density. Lagoons were filled with DRM and constantly diluted with culture from the chemostat following initiation of the pumps. Lagoons were allowed to equilibrate for ~8 h prior to the addition of phage. Unless stated otherwise, lagoons were maintained at a volume of 20 mL and fed at a flow rate of 1 vol h^−1^ from the chemostat. Any changes in dilution rate during PACE experiments were exclusively implemented through alterations to pumping rate, rather than adjusting lagoon volume. For PACE experiments involving the inducible BCM efflux pump plasmid pPUMP, chemostat DRM was modified with carbenicillin for plasmid maintenance and 5 μM IPTG for constitutive induction.

Following equilibration of lagoon and chemostat cultures, lagoons were seeded with starting titers of ~10^6^ pfu mL^−1^ and grown for 12–16 h overnight without mutagenesis to reach high titers for the initiation of mutagenic PACE the following morning. After overnight culturing, lagoons were sampled prior to the addition of mutagen (*t* = 0 h) by removing 3 mL of culture via sterile syringe. For mutagenesis, MNNG (TCI America) was dissolved in DMSO for direct infusion and dilution into lagoons, pumped from 50 mL syringes through Versilon SE-200 chemical transfer tubing (Saint-Gobain) using a syringe pump (New Era Pump Systems) at a flow rate between 0.1 and 1.0 mL h^−1^. Following the initiation of mutagen addition, lagoons were sampled at regular intervals (roughly every 12 h).

Samples taken from lagoons were processed immediately to isolate phage particles and dsDNA. One milliliter of culture sample was transferred to a 1.7 mL Eppendorf tube, centrifuging at 2000 *g* for 5 min. Supernatants were filtered using sterile polyethersulfone 0.2-μm centrifuge filters (mdi Membrane Technologies), centrifuging again at 2000 *g* for 5 min. Samples were stored at 4 °C for up to 1 week before use. The remaining culture sample was centrifuged in the original 1.7 mL Eppendorf tube at 20,000 *g* for 1 min to collect the cell pellet for plasmid isolation. Genotype assessment and monitoring of the integrity of the BCM operon was performed regularly by PCR off of isolated phage dsDNA using primers BCM_CWJ_USER_F (5′-AGGGGCCCAAG_d_UTCACTTAAAAAGGAG) and BCM_CWJ_USER_R (5′-ACTCTAGATCAATA_d_UA-GACCCTGGGTATTCTC) and a standard thermocycler program (98 °C 1 min, followed by 30 cycles of 98 °C for 10 s, 60 °C for 30 s, 72 °C for 3 min 30 s, followed by 72 °C for 10 min). PCR products were assessed by 0.8% agarose gel electrophoresis. For operon recovery, the resulting PCR product was gel excised and cloned into an inducible plasmid backbone. Assembled BCM EPs were transformed into NEB Turbo cells carrying a fluorescent reporter and plated on 2× LB agar containing appropriate antibiotic and 100 ng mL^−1^ aTC, growing at 30 °C for 48–96 h. GFP expression from individual colonies was monitored via inspection on a SafeImager 2.0 UV imager (Invitrogen). Colonies that expressed GFP were isolated, retested in liquid culture, and grown for plasmid isolation and Sanger sequencing (Quintarabio) to determine genotype.

Phage titers were determined by plaquing filtered culture supernatant samples on S2060 cells carrying the activity-independent permissive plasmid pJC175e^11^. Samples were checked periodically for the presence of recombinant phage bearing pIII by plaquing on S2060 without pJC175e.

### Statistics and reproducibility

All assays and LC–MS quantification presented in this work were repeated independently at least three times and provided reproducible results.

### Reporting summary

Further information on research design is available in the Nature Research Reporting Summary linked to this article.

## Supplementary information

Supplementary Information

Peer Review File

Reporting Summary

Description of Additional Supplementary Files

Supplementary Data 1

Supplementary Data 2

Supplementary Data 3

## Data Availability

Data supporting the findings of this study are available within the paper and its Supplementary Information files. A reporting summary for this article is available as a Supplementary Information file. The datasets generated and analyzed during the current study (including LC–MS data files of Fig. 2g and Supplementary Figs. 10, 11 and 12) are available from the corresponding author upon request. Source data are provided as a Source Data file.

## Source data

Source Data
