# Supplementary material for: Continuous bioactivity-dependent evolution of an antibiotic biosynthetic pathway
Source: Nat Commun. 2020 Aug 21;11:4202. doi: 10.1038/s41467-020-18018-2 (PMC7443133; doi:10.1038/s41467-020-18018-2)
Supplement: Supplementary file 1 — Supplementary Information [file 41467_2020_18018_MOESM1_ESM.pdf]

Continuous bioactivity-dependent evolution of an antibiotic biosynthetic pathway

Johnston *et al.*

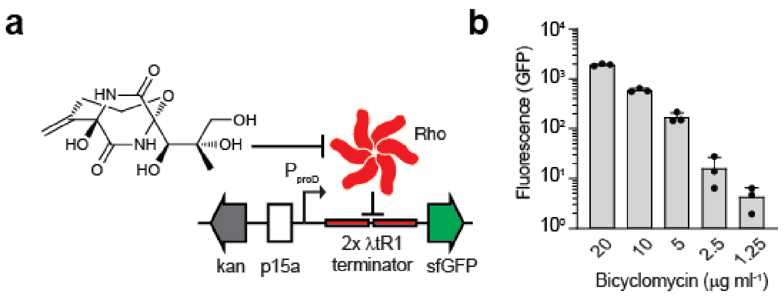

Supplementary Fig 1. High-stringency bicyclomycin reporter plasmid pREP.2. a, Schematic for alternative, high-stringency bicyclomycin (BCM) reporter plasmid pREP.2, featuring two copies of the Rho-dependent λtR1 terminator which silence superfolder GFP (sfGFP) expression in the absence of BCM. **b**, pREP.2 dose-dependent response to BCM. *E. coli* cells carrying pREP.2 were grown in 2x LB with antibiotics at 37°C with shaking to stationary phase, then diluted 1:100 into fresh media and grown in identical conditions until OD600nm of 0.6-0.8. Cells were diluted 1:100 into a deep 96-well plate, containing fresh media warmed to 30°C, with/without a serial dilution of BCM. Cells were grown for 12 h with shaking at 30°C. Results are absorbance normalized signal over no drug controls, shown as mean ± s.d.; n = 3 biological replicates. Source data underlying Supplementary Figure 1b are available as a Source Data file.

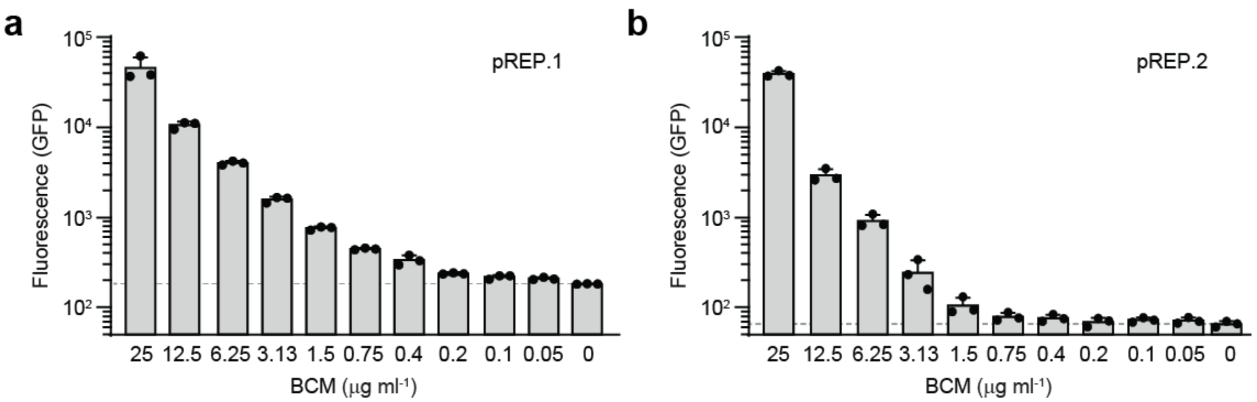

Supplementary Fig 2. Comparison of BCM reporter plasmids pREP.1 and pREP.2. Dose-dependent responses of BCM reporter plasmids pREP.1 (**a**) and pREP.2 (**b**) to BCM. *E. coli* cells carrying reporter plasmids were grown in 2x LB with antibiotics at 37°C with shaking to stationary phase, then diluted 1:100 into fresh media and grown in identical conditions until OD600nm of 0.6-0.8. Cells were diluted 1:100 into a deep 96-well plate, containing fresh media warmed to 30°C, with/without a serial dilution of BCM. Cells were grown for 12 h with shaking at 30°C. Results are absorbance normalized signal, shown as mean ± s.d.; n = 3 biological replicates. Signal from a no-drug control is marked with a dashed line. Source data are available as a Source Data file.

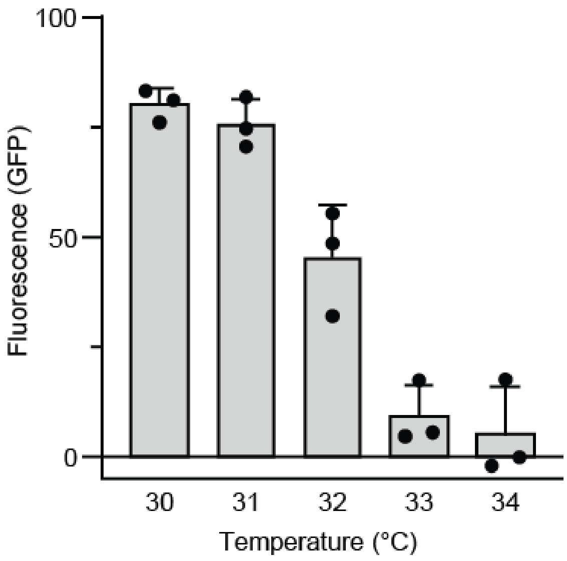

Supplementary Fig 3. Temperature sensitivity of BCM biosynthesis. *E. coli* cells carrying BCM EP pBCM.1 and pREP.1 were grown to stationary phase at 37 °C with shaking, followed by 1:100 dilution into fresh media and growth to late exponential at 37°C with shaking. Cells were diluted 1:100 into fresh media warmed to an appropriate temperature with antibiotics and with/without 100 ng ml^{-1} anhydrotetracycline (aTC), then grown with shaking at the stated temperature (30°C to 34°C) for 12 h. Fluorescence and absorbance were measured after incubation. Results are absorbance normalized signal over uninduced controls, shown as mean ± s.d.; n = 3 biological replicates. Source data are available as a Source Data file.

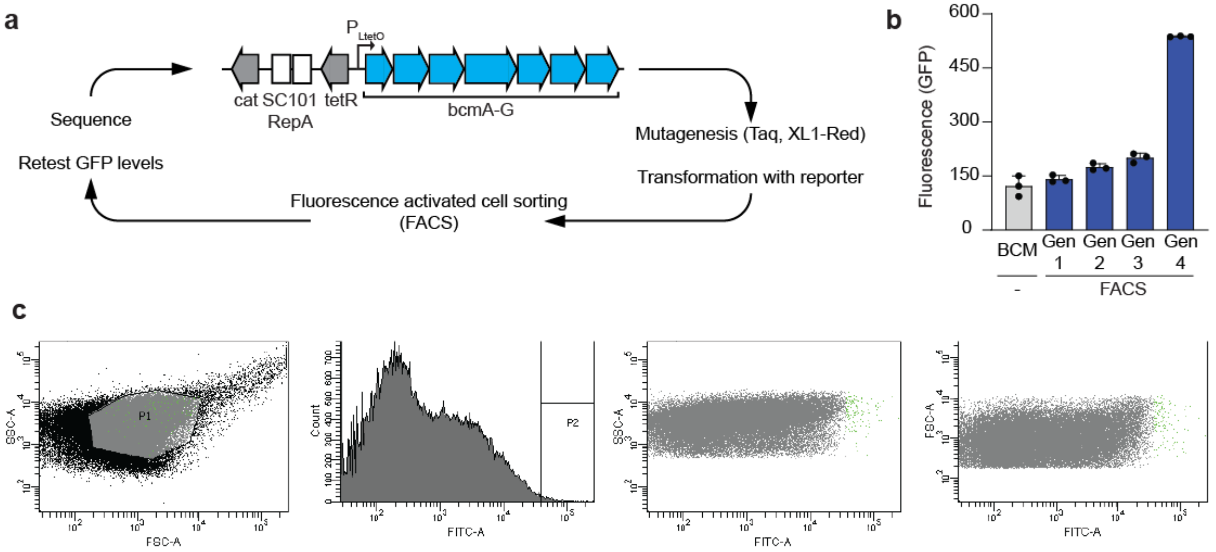

Supplementary Fig 4. Evolution of BCM EP with FACS selection. **a**, Non-continuous evolution scheme for BCM expression plasmid (EP). Mutations were introduced into BCM EP through amplification of the BCM operon with low-fidelity Taq DNA polymerase or through serial passaging of BCM EP in mutagenic XL1-Red *E. coli*. Mutagenized BCM EP was transformed into *E. coli* carrying pREP.1 and induced prior to selection sorting via FACS. Sorted fluorescent cells were recovered on LB agar plates with appropriate antibiotics, retested for GFP production following BCM EP induction, and prepped for plasmid isolation and sequencing. The most active clone of each round was used for the next round of evolution. **b**, *E. coli* cells carrying pREP.1 and BCM expression plasmids from sequential rounds of FACS evolution (wildtype: BCM; Taq

mutagenesis: Gen 1; XL1-Red mutagenesis: Gen 2-4) were grown to stationary phase at 37°C with shaking, followed by 1:100 dilution into fresh media and growth to late exponential at 37°C with shaking. Cells were diluted 1:100 into fresh media warmed to 30°C with antibiotics, with/without 100 ng ml^{-1} anhydrotetracycline (aTC), and grown with shaking at 30°C for 12 h. Fluorescence and absorbance were measured after incubation. Results are absorbance normalized signal over uninduced controls, shown as mean ± s.d.; n = 3 biological replicates. **c**. Representative gating strategies for FACS selection, including forward and side-scatting gate P1 and fluorescence (GFP; FITC) gate P2. Source data underling Supplementary Figure 4b are available as a Source Data file.

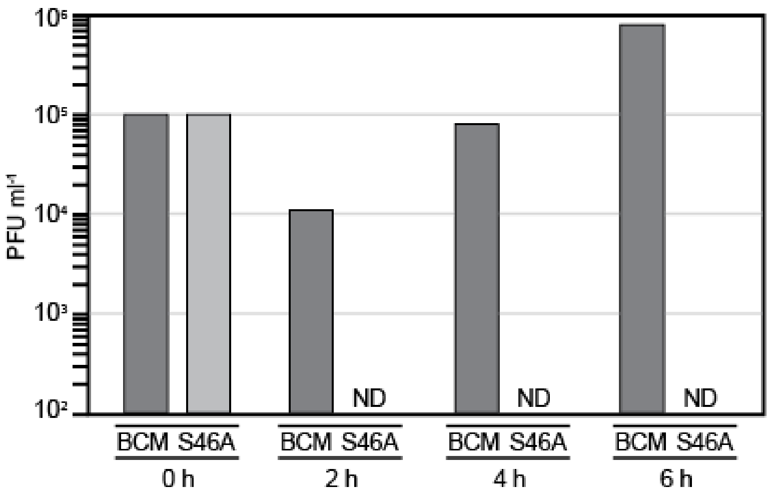

Supplementary Fig 5. Propagation of wildtype and BcmA S46A SP$_{BCM}$. *E. coli* S2060 cells carrying accessory plasmid pAP.1 were grown in 4 ml of Davis rich media (DRM) at 30°C until reaching OD600nm = 0.6-0.8. Cultures were split into two identical 2 ml cultures, and selection phage (SP) bearing the FACS evolved BCM operon (SP$_{BCM}$) or the inactive BcmA S46A BCM operon (SP$_{S46A}$) were added to a final concentration of 10^5 PFU ml^{-1}. Following SP addition, phage infect *E. coli* cells and can produce infectious progeny if they pass the BCM-dependent genetic circuit of pAP.1. Supernatant samples of 100 µl were taken after 2, 4, and 6 h, centrifuged, and filtered to isolate phage. Samples were plaqued on *E. coli* 2060 cells carrying the permissive, activity-independent plasmid pJC175e[1] to provide PFUs. No plaques were detected (ND) from the catalytically inactivated SP$_{S46A}$. Source data are available as a Source Data file.

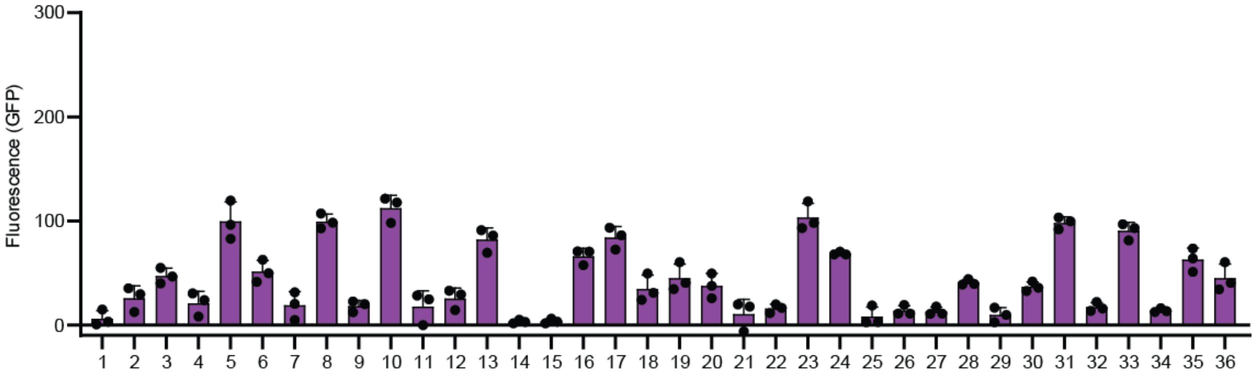

Supplementary Fig 6. BCM operons recovered from 72 h PACE. *E. coli* cells carrying pREP.2 and BCM expression plasmids with BCM BGC sequences recovered after 72 h PACE evolution (72.1 to 72.36; Supplementary Data Set) were grown to stationary phase at 37°C with shaking, followed by 1:100 dilution into fresh media warmed to 30°C with antibiotics, with/without 100 ng ml^{-1} anhydrotetracycline (aTC), and grown with shaking at 30°C for 12 h. Fluorescence and absorbance were measured after incubation. Results are absorbance normalized signal over uninduced controls, shown as mean ± s.d.; n = 3 biological replicates. Source data are available as a Source Data file.

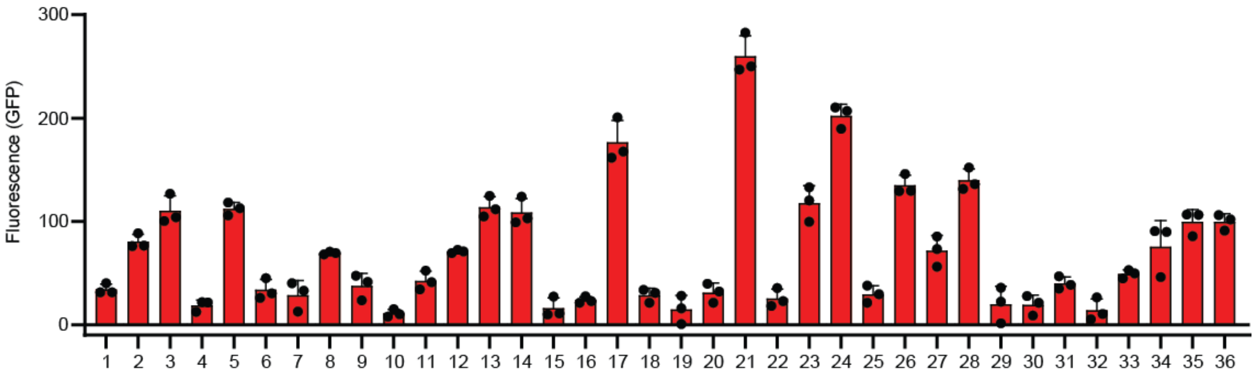

Supplementary Fig 7. BCM operons recovered from 144 h PACE. *E. coli* cells carrying pREP.2 and BCM expression plasmids with BCM BGC sequences recovered after 144 h PACE evolution (144.1 to 144.36; Supplementary Data Set) were grown to stationary at 37°C with shaking, followed by 1:100 dilution into fresh media warmed to 30°C with antibiotics, with/without 100 ng ml^{-1} anhydrotetracycline (aTC), and grown with shaking at 30°C for 12 h. Fluorescence and absorbance were measured after incubation. Results are absorbance normalized signal over uninduced controls, shown as mean ± s.d.; n = 3 biological replicates. Source data are available as a Source Data file.

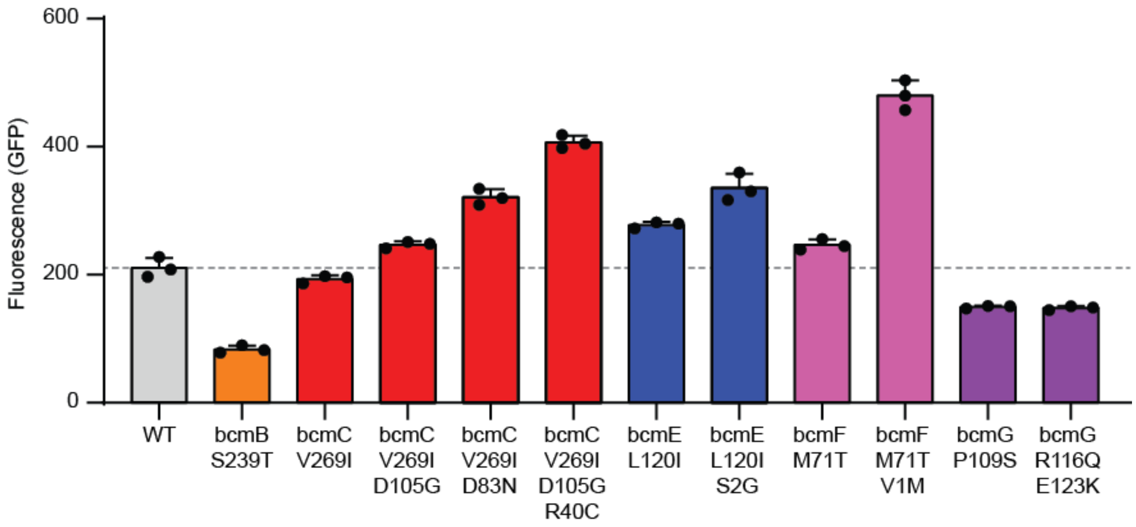

Supplementary Fig 8. Impact of evolved mutations on BCM production. Notable mutations observed during BCM operon evolution were cloned individually into a wildtype (WT) inducible BCM production plasmid (pBCM.1) and expressed in *E. coli* cells carrying reporter plasmid pREP.1. *E. coli* cells were grown to stationary at 37°C with shaking, followed by 1:100 dilution into fresh media warmed to 30°C with antibiotics, with/without 100 ng ml^{-1} anhydrotetracycline (aTC), and grown with shaking at 30°C for 12 h. Fluorescence and absorbance were measured after incubation. Results are absorbance normalized signal over uninduced controls, shown as mean ± s.d.; n = 3 biological replicates. Source data are available as a Source Data file.

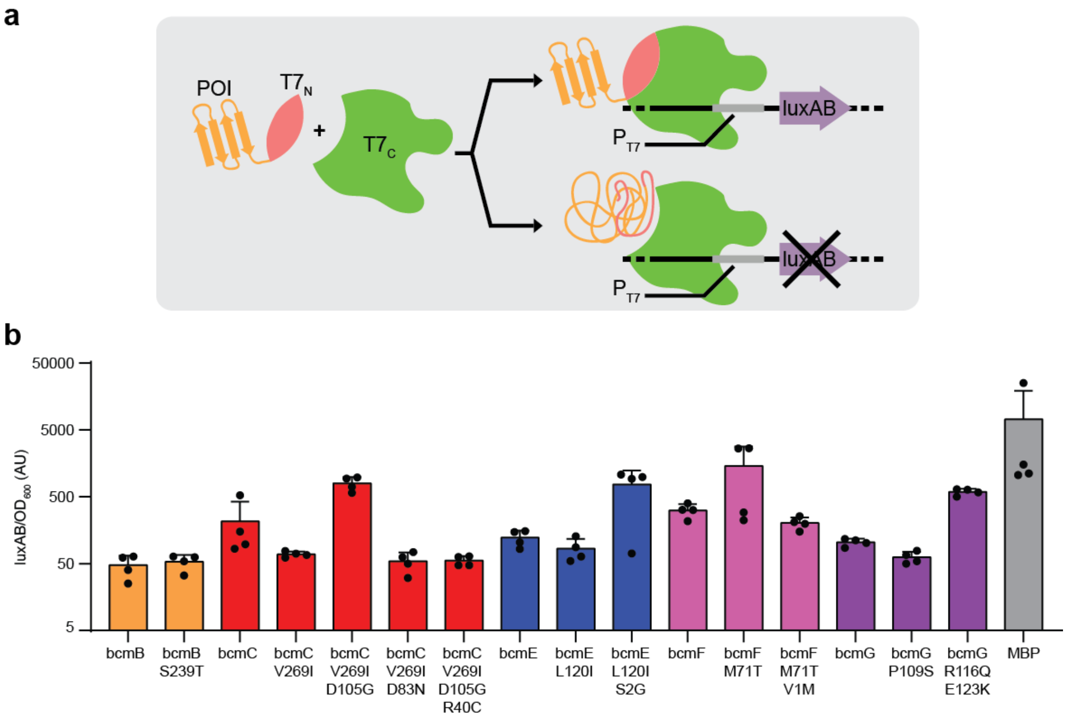

Supplementary Fig 9. Impact of evolved mutations on soluble protein expression. **a,** Schematic of luminescence reporter assay for assessing protein soluble expression. Bicyclomycin biosynthetic proteins of interest are fused to the N-terminal fragment of T7 RNA polymerase. Soluble protein-of-interest (POI) fusions complement the C-terminal T7 RNA polymerase fragment, resulting in *luxAB* expression. Schematic and assay adapted from Wang *et al*.[2] **b**, Impact of evolved mutations on soluble protein expression. Mutations are shown corresponding to the order in which they were first observed during evolution. In several cases, initial improvements in soluble expression facilitated access to mutations that increased BCM production but lowered soluble expression (BcmC R40C, BcmF V1M). The highly soluble maltose-binding protein (MBP) was used as positive control. Results shown as mean ± s.d.; n = 4 biological replicates. Source data are available as a Source Data file.

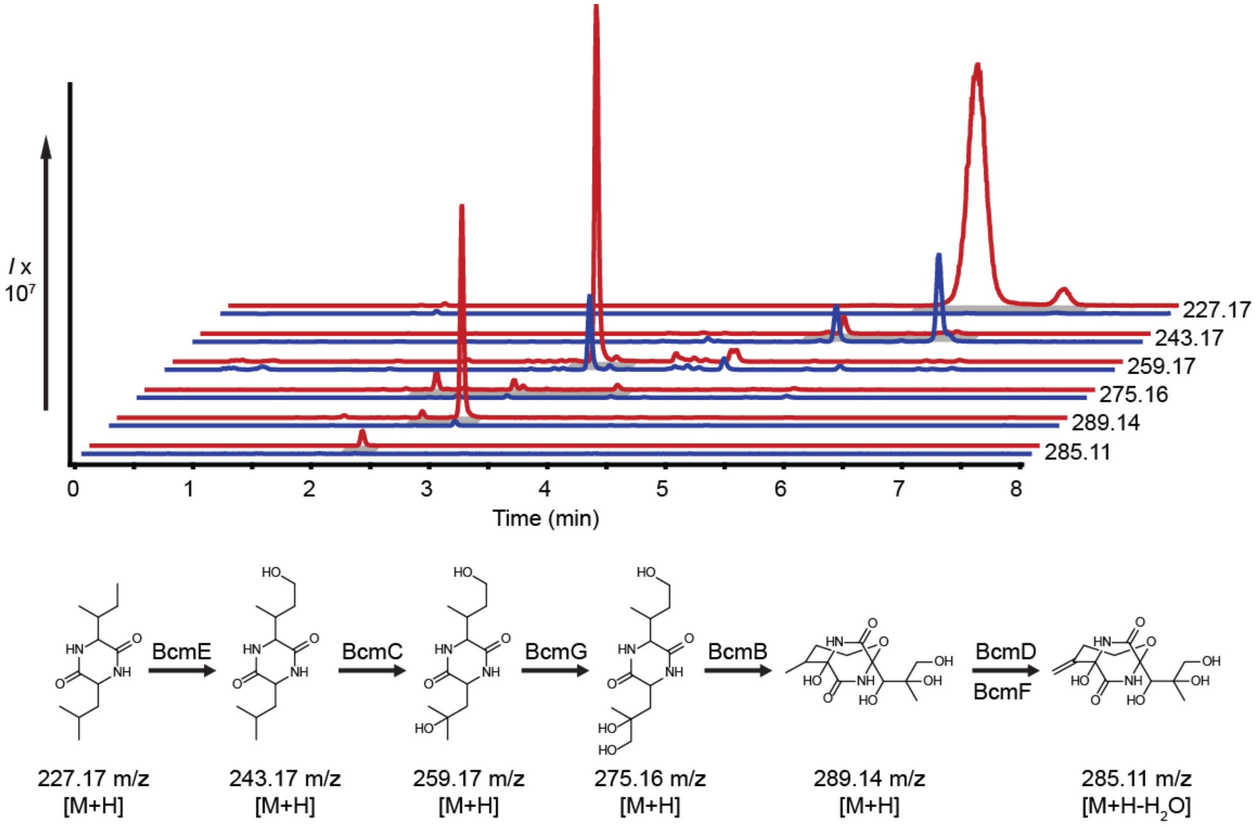

Supplementary Fig 10. Metabolomic comparison of wildtype and evolved BCM operons in *E. coli*. Liquid chromatography and mass spectrometry (LC-MS) analysis of culture supernatants from *E. coli* carrying induced BCM production and efflux plasmid pBCM.2, bearing wildtype (blue) or evolved (red; PACE clone 72.5) BCM operons. Extracted ion chromatograms of observed BCM metabolites revealed increased levels of nearly all intermediates from the evolved operon, with the exception of the singly hydroxylated cyclo-Ile-Leu (243.17 m/z [M+H], which was more abundant in wildtype supernatants.

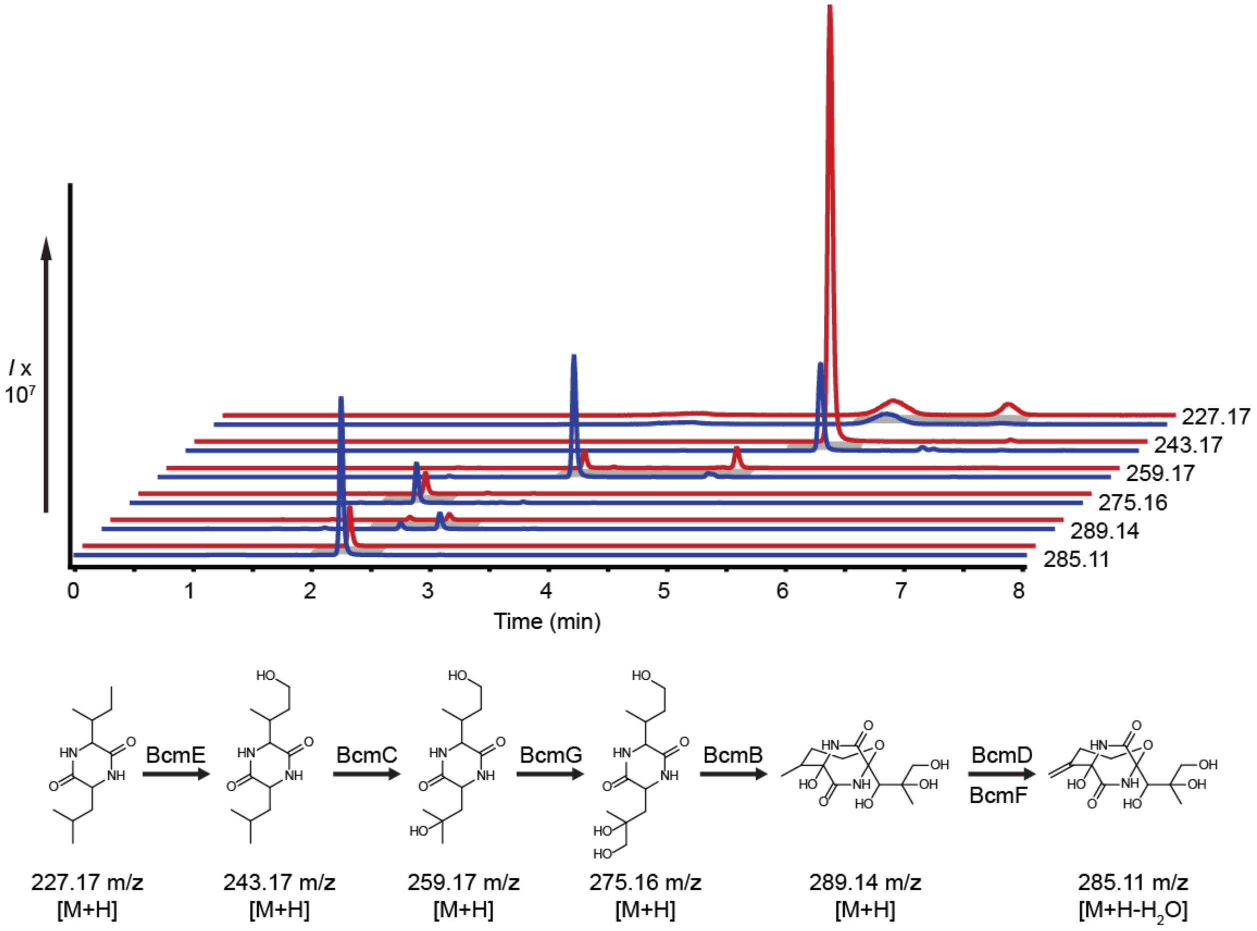

Supplementary Fig 11. Metabolomic comparison of wildtype and evolved BCM operons in *P. fluorescens*. Liquid chromatography and mass spectrometry (LC-MS) analysis of culture supernatants from *P. fluorescens* SBW25 carrying BCM production and efflux plasmid pJH.BCM[3], bearing wildtype (blue) or evolved (red; PACE clone 72.5) BCM operons. Extracted ion chromatograms of observed BCM metabolites revealed higher levels of nearly all intermediates from the wildtype operon, with the exception of the singly hydroxylated cyclo-Ile-Leu (243.17 m/z [M+H], which was more abundant in evolved supernatants.

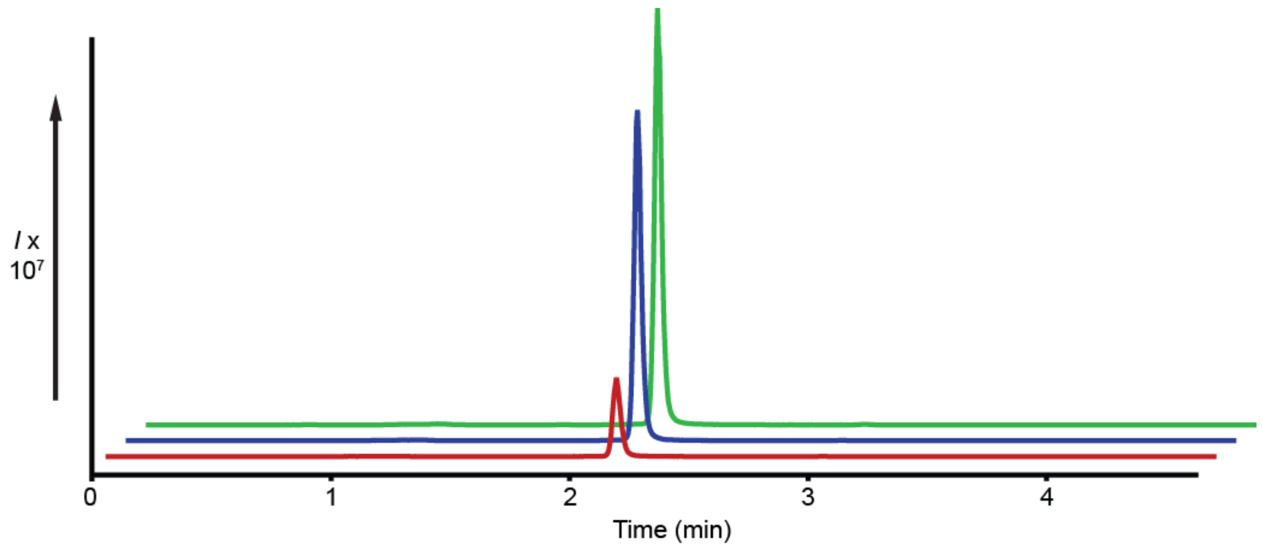

Supplementary Fig 12. Comparison of BCM yields from different operons expressed in *P. fluorescens*. Extracted ion chromatograms for BCM (m/z = 285.1 [M+H-H_2O]) taken from LC-MS data of culture supernatants from *P. fluorescens* SBW25 carrying BCM production plasmid pJH.BCM[3], containing our evolved operon (red; PACE clone 72.5), our wildtype operon (blue), or a previously published wildtype BCM operon[3] (green).

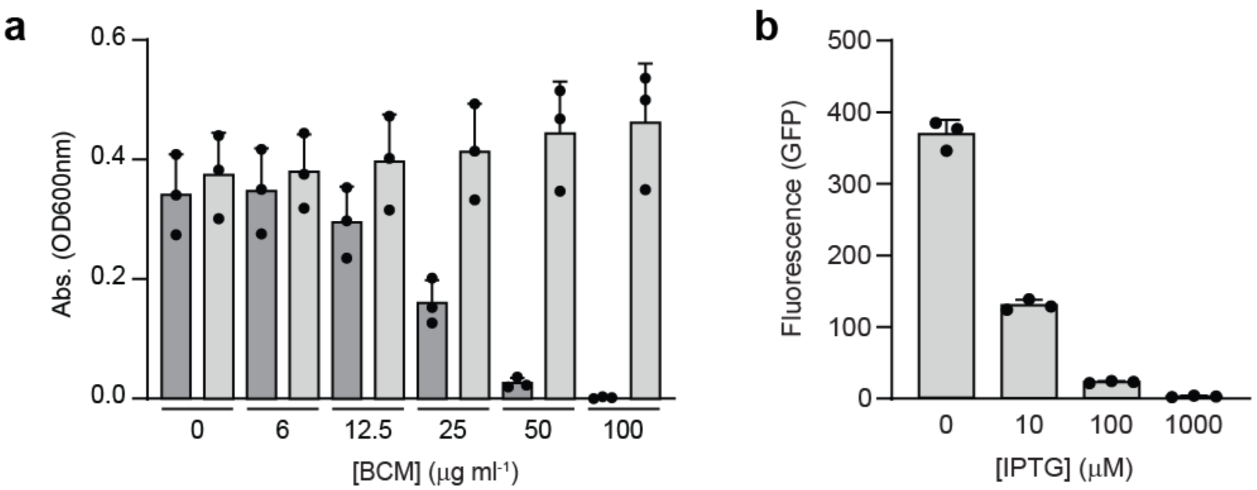

Supplementary Fig 13. Active efflux of BCM reduces signal from the BCM reporter. **a,** Optical density of *E. coli* cultures carrying the inducible BCM efflux pump plasmid pPUMP, with (light grey) or without (dark grey) induction by 1 mM IPTG, grown in a serial dilution of BCM. **b**, Fluorescence (GFP) signal from BCM reporter plasmid pREP.2, following exposure to 10 μg ml^{-1} BCM, in *E. coli* cells carrying pPUMP. *E. coli* cells were grown to stationary at 37°C with shaking, followed by 1:100 dilution into fresh media warmed to 30°C with antibiotics, BCM, with varying concentrations of IPTG, then grown with shaking at 30°C for 12 h. Fluorescence and absorbance were measured after incubation. Results are shown as mean ± s.d.; n = 3 biological replicates. Source data are available as a Source Data file.

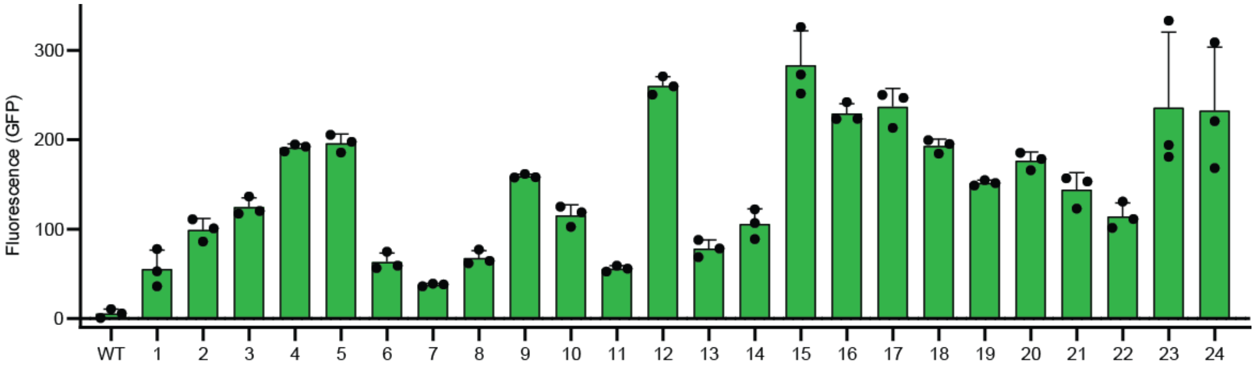

Supplementary Fig 14. BCM operons recovered from an additional 72 h PACE with efflux. *E. coli* cells carrying pREP.2 and BCM expression plasmids with BCM BGC sequences recovered after an additional 72 h PACE evolution (Pump.1 to Pump.24, with wildtype (WT) control; Supplementary Data Set) were grown to stationary at 37°C with shaking, followed by 1:100 dilution into fresh media warmed to 30°C with antibiotics, with/without 100 ng ml^{-1} anhydrotetracycline (aTC), and grown with shaking at 30°C for 12 h. Fluorescence and absorbance were measured after incubation. Results are absorbance normalized signal over uninduced controls, shown as mean ± s.d.; n = 3 biological replicates. Source data are available as a Source Data file.

Supplementary Table 1. Plasmids constructed and used in this study.

Name	Class (res)	Origin	Prom.	Feature.	RBS.	gene(s).	Use
pBCM.1	Expression (chlor)	SC101 RepA E93K	P_L	tetO	BCD13	bcmA-G	BCM expression plasmid
pBCM.2	Expression (chlor)	SC101 RepA E93K	P_L	tetO	BCD13	bcmA-G bcmT	BCM expression plasmid with efflux
pAP.1	AP (kan)	SC101	P_{proB}	2x λtR1	SD8	gIII	BCM-dependent gIII expression
pREP.1	Expression (kan)	pBR322	P_{J23101}	1x λtR1	SD8	SFGFP (ASV deg)	BCM fluorescent reporter (relaxed)
pREP.2	Expression (kan)	p15a	P_{proD}	2x λtR1	SD8	SFGFP (ASV deg)	BCM fluorescent reporter (stringent)
pJH.BCM[3]	Expression (tet)	RSF1010	P_{tac}	-	native RBS	bcmA-G bcmT	BCM expression plasmid with efflux in *P. fluorescens*
pPUMP	Expression (spec)	ColE1	P_{lacZ}	lacIO	SD8	bcmT Efflux pump	Inducible BCM efflux
			P_{laciq}	-	lacI RBS	lacI	
SP_{BCM}	SP (none)	M13 f1	P_{gIII}, P_L	-	BCD13	bcmA-G	BCM operon evolution
SP_{S46A}	SP (none)	M13 f1	P_{gIII}, P_L	-	BCD13	bcmA-G (bcmA S46A)	Phage enrichment negative control
pTW004[19]	Expression (spec)	ColE1	P_L	tetO	SD8	T7n fusion	Complement T7c, measure soluble expression
pTW006[19]	Expression (carb)	SC101	P_{T7}	-	SD8	luxAB	Luciferase reporter for soluble expression of T7n
			P_{BAD}	lacIO	SD8	T7c	
			P_C	-	SD8	araC	

Supplementary References

1. Carlson, J.C., Badran, A.H., Guggiana-Nilo, D.A. & Liu, D.R. Negative selection and stringency modulation in phage-assisted continuous evolution. *Nat Chem Biol* **10**, 216-222 (2014).
2. Wang, T., Badran, A.H., Huang, T.P. & Liu, D.R. Continuous directed evolution of proteins with improved soluble expression. *Nat Chem Biol* **14**, 972-980 (2018).
3. Vior, N.M., *et al*. Discovery and biosynthesis of the antibiotic bicyclomycin in distantly related bacterial classes. *Appl Environ Microbiol*. **84**, e02828-17 (2018).
