# Supplementary material for: Continuous bioactivity-dependent evolution of an antibiotic biosynthetic pathway
Source: Nat Commun. 2020 Aug 21;11:4202. doi: 10.1038/s41467-020-18018-2 (PMC7443133; doi:10.1038/s41467-020-18018-2)
Supplement: Supplementary file 4 — Description of Additional Supplementary Files [file 41467_2020_18018_MOESM4_ESM.pdf]

Description of Additional Supplementary Files

File name: Supplementary Data 1
Description: Summary of mutations observed following PACE experiments

File name: Supplementary Data 2
Description: Sequence alignments of mutations observed following experiments

File name: Supplementary Data 3
Description: Primers used in this study
