# Supplementary material for: Continuous bioactivity-dependent evolution of an antibiotic biosynthetic pathway
Source: Nat Commun. 2020 Aug 21;11:4202. doi: 10.1038/s41467-020-18018-2 (PMC7443133; doi:10.1038/s41467-020-18018-2)
Supplement: Supplementary file 6 — Supplementary Data 2 [file 41467_2020_18018_MOESM6_ESM.docx]

**Supplementary Data 2. Sequence alignments of observed bcm variants**

**Organisms associated with observed sequence variations**

*bcmA*: *Pseudomonas aeruginosa* N17-1

*bcmA* L14I: *Burkholderia plantarii*

*bcmA* P91S: *Sphingobium* sp.

*bcmA* V237I: *Providencia heimbachae*

1 10 20 30 40 50 60

| | | | | | |

bcmA ------MNLIAEDFSTRSL**L**AGERYKAKIAFVSPHTRRNSFEDEPKCFLGVSLENRNFTP

bcmA L14I MRLKVTIDETLIGNLPISS**I**PGARYKAKIDFVSPQSRRDNFQLEQQCFLGVSLENRNFEP

bcmA P91S ------MNLYSDILDRPSSTPLAKYRAKIAFVSPPGRRDSFEQEARCFVGVSLENRNFEP

bcmA V237I -------MSKLENIKNNEPATKKKYHAKTNIVHPIFNRKNFESQEECFLGISLENKNFVP

bcmA NRFHSMVEWAARRFEKCSILIGDHIHRITLQSTRDM**P**EEDARREAVRLGDAFMEESRLIV

bcmA L14I ARFHSQLEWVSRRFPKCTILIGDSIHRLTLESRSGMPAQEAFDRALQLGQEFMEESRDIV

bcmA P91S ARFYAQLEWAARRFSDVQILVGDGIHRISVETVHGL**S**PRQAEERALELGRSFMRDNAHLL

bcmA V237I EKFIAMAKWINQRFKRCHILIGDSIHRITLETNKKYTPEHAFPEAIKLGEAYRDQAIAQL

bcmA DAYRHATDFQYITCSEIQASTECKDFYSSLRNYFLEHSGFRDSVEKFGRNYHRHNWEKLS

bcmA L14I TDYRATTRFEFITCHEIQKTQEYLMHHRNISDYFFQSRKFRESVESFGLRYHRNDWDTLT

bcmA P91S ASFQDMAQINYLTCSAIQDIPSYSAFLSKLWGYFDRVPSFRQSVEAFAAHYHRKNWDQLS

bcmA V237I ELYKFQTEFSFVTCYSLQQTPEYQNFHSNIDHFFYFSKKFRESVEKFSLIFHRYHWDNLN

bcmA EEQQRHRLDFSSRYFLEEFSVFACLVRKGFKVMAYPGAFSTLAEIAAGDFPGMLDELQKL

bcmA L14I RAEQDSRLRSSSNYFIEEFAIFACLVKRGFSVMVYPGSFSTLAEIADHQFPGVSKELESL

bcmA P91S ERDRAYRLKRSCEYFLEEFAIFACLVERGNSVMVYPGSFSTLAEIANGDFPGIPPELEAL

bcmA V237I ELQRKYCIDKSSQYFLEEFSIFACLVKKGINVMVYPGTFGPLTEIVNGEHNGIIDELENL

bcmA TV**V**SLQLKRR----------------

bcmA L14I CVVSLHFKGR----------------

bcmA P91S TVVSLQLKKR----------------

bcmA V237I TV**I**SLNIKNVNKSKSRGFINEWKYIK

**Organisms associated with observed sequence variations**

*bcmC*: *Pseudomonas aeruginosa* N17-1

*bcmC* D83N: *Photorhabdus bodei*

*bcmC* D105G: *Pseudomonas psychrophila*

*bcmC* P183S: *Pseudomonas psychrophila*

*bcmC* E237K: *Beijerinckia indica subsp. indica*

*bcmC* P250S: *Mycobacteroides chelonae*

*bcmC* V269I: *Photorhabdus khanii*

1 10 20 30 40 50 60

| | | | | | |

bcmC ------------------------MSGLPQFHPCKAYLDDSRHLVFNQADGFARALRDGF

bcmC D83N MKTSS--TTRAPVKLPPMPSESEASKTYKSADMARSYLEGD-NLIFTDNDGFNRALRDGF

bcmC D105G -------------------------MKTHKINMQRAIIYQD-ALLFKTPFSLRAAIEQGL

bcmC P183S -------------------------MKTHKINMQRAIIYQD-ALLFKTPFSLRAAIEQGL

bcmC E237K -------------------------MVLQSFLPARAKFHDG-QLTFVVGD-LGTALRQGC

bcmC P250S MTTSNPVTARAPVSLPPMPREHEALAAYPPIQLERAHLDGE-QLLFDSAEGFDRALAQGF

bcmC V269I -------------------------MTLSIFVPSRARIDEN-RIVFSSGN-MVTALRDGC

bcmC FALRIPEELDLAPGIRFAQEFYQPAVEEPHA---DARYRGFRNLPDIYF**D**RENFQTEHIL

bcmC D83N FLLKAPPYMDFKYGDRFAYHFFEPATEGDLRPYTGFKSYTFSNEYEGYF**N**REHDQWEQFR

bcmC D105G FLLKIPETLNLEPGIKLCREFYLPATS-------STRYRGFRPQTDIYFDREHFQTEHLL

bcmC P183S FLLKIPETLNLEPGIKLCREFYLPATS-------STRYRGFRPQTDIYFDREHFQTEHLL

bcmC E237K FALRIPDELDLAPGIRLAQEFYLAG-------------RGVQDREGIYFDQELFQTDHVL

bcmC P250S FLLRIPEALDTEPGDRFAAHFYQDRAGDELDRYRGFREIDVPGTYQGYFDREHDQWENFY

bcmC V269I LALQIPDGLDLAPGKKLAREFYLDPITYSNL---SIGYRGFRYRSDIYFDREHFQTEHIL

bcmC ADARQRQASFP**D**EVNRLCERMHEIARLILREILGSLGVAPRLWPDVTGGTSEGKGVTWFA

bcmC D83N LEREHWN-LMPSEVATLGHHMAHVGICVLRSVLTYVGIPRHDWELVTNGISEGLGNQMLS

bcmC D105G VDKTRWATRLP**G**AVVKMVLAMDALGQVILQAALRELGVAQALWASASGGAVTGQGTHWFA

bcmC P183S VDKTRWATRLP**G**AVVKMVLAMDALGQVILQAALRELGVAQALWASASGGAVTGQGTHWFA

bcmC E237K IDGPRRRKDFPQAVNLMCEKMCALARFLLRIILTEAGISESLWADATDDCVTDGGVQWFA

bcmC P250S IEQANWA-LLPADVAELGRGMTELGIAVLRAVFRRLRIPAELWSQVSSGLSENRGHQMLA

bcmC V269I ADELQRKSTFPTDVNSMCGKMHDLARIVLQEILFDIGVSPLLWNKVTDSATKGGGIKWFA

bcmC VSHYRPERNMQGAPAHKDTGFVTVLYCDQ**P**GLQARLEEGWVEVPPMEGHFLINFGGSLEL

bcmC D83N FNHFRSEKATRGSKFHRDSGWVTVLRSTERGLLAYINDELLAIDPVPGYLIINFGSSIEV

bcmC D105G ASHYRPERHLPGCAEHQDTGFVTLLYTRQ**S**GLEAFIDGGWQPIEPEPGYFIVNFGACLEL

bcmC P183S ASHYRPERHLPGCAEHQDTGFVTLLYTRQ**S**GLEAFIDGGWQPIEPEPGYFIVNFGACLEL

bcmC E237K LGHYQSDRNLPGAPAHKDPGFITVLYYDKPGLEAYIDGEWRNIPPVGGYFLINFGGALEI

bcmC P250S FNHFRSQKTTRGCKFHRDSGWVTVLRSVDPGLLALIEGRLGSINPERGYFIVNFGSSLEV

bcmC V269I VNHYRPNRQKPGAPAHKDTGFITVLFCEQPGLEALINDQWMSIEPIGGYFLINFGGTLEL

bcmC LTARLPIPAAAVLHQVRQSSSTV**E**RGDRYSFAAFLN**P**PATGALYSLTADGQRAEP**V**IPVE

bcmC D83N LTENLIPNVRANIHGVARTERNR-QCDRMSYVMFLV**S**DLAGNVYKYGPNG-PIK-VQSVK

bcmC D105G LSAQLPLSARAIKHRVRECPSLPGQEDRFSFATFINPPASADLYEVQPLG-ELRRLMSAE

bcmC P183S LSAQLPLSARAIKHRVRECPSLPGQEDRFSFATFINPPASADLYEVQPLG-ELRRLMSAE

bcmC E237K LTAKCSVPVKAVLHRVRQCLPAS**K**TGDHFSFAVFLNPAADGDVIQASADGKTLVSLGSVD

bcmC P250S LTADLPTPVRANIHGVVGTERAAGQPDRISYVTFLD**S**NLDGTIYRITNDG-TAQPVQSVA

bcmC V269I ITKNLPTKVNAVLHRVKQCEFNPELGDRYSFAAFINPSASCDIFQVSEDGQEAIP**I**TSVE

bcmC TFLREFNEQTWKDSYTDFGIAEGPGAAAPADDHTNRYLRP--

bcmC D83N EFVIQEASRTYDDDNDTL------------------------

bcmC D105G AFLRRFNERTWQDRHASFGILSEQTQGTPNE-----------

bcmC P183S AFLRRFNERTWQDRHASFGILSEQTQGTPNE-----------

bcmC E237K EYLRTLNKMTWHDGYAAFGITRKSHDMVLVPQTCDHPVKGPR

bcmC P250S DFAVQEVNRTYNDDITL-------------------------

bcmC V269I EFLRNFNKQTWQDNYSDFGITKKQVNTLSENNNNQR------

**Organisms associated with observed sequence variations**

*bcmD*: *Pseudomonas aeruginosa* N17-1

*bcmD* L130M: *Sphingobium* sp.

*bcmD* M223I: *Burkholderia plantarii*

1 10 20 30 40 50 60

| | | | | | |

bcmD --------MNSKENSAPFPGLIAHHSGSCPATGHAEAFHHATVCQGLDASTYFARSGIAE

bcmD L130M MSASSRAAPVGRSRTAPFPGIIAHLAGPDPDGGHEKAFHIATVEQGMDASAYFEQAGFPA

bcmD M223I MTIPTLIPPSQHTRAVPFPGLIAHAPGPDTDTEHGAAFRAAVIEGGLDASDYFARSGVAE

bcmD LAENNEGLCTFWLGDDLALYQTTNAPLVDDEDLAPSINANAELFGSFLGSLPAQDERRKA

bcmD L130M LAERSGGLCSFWLGANLALYQSINDPLVNDSDLAPSINANADLFGSFMGALDIDDPTRAG

bcmD M223I LASRSGGLCTLWIDNDLAIYQNTNEPLVEDDDLMPSINTNAGLFGSFMGGLDANDPVRVA

bcmD KRAVVERVLGSNRFVTS**L**DPHVREMAQHYLREVAGRSLPLQDFCLHMVARIDSGLPGVLD

bcmD L130M RRAVVERVLGSAKFINA**M**EAEASQIIGEYLAKVEGRELPMDEFALNLTAYVDSLVPGVLD

bcmD M223I KREFVERILGNARFVYGLSDEITAATRAYLDSPLSQDTSLDTFCLNLTAYVDSVIPGILD

bcmD FHQKPLTHYLQSTEYGVIARDFFEIASEVISKMNPESIENADMIVEMTRD**M**LDSNYESIV

bcmD L130M FRNVPLTDYLDSDQYGRIARSFFEIASEVISKLSPEAIKDADLIVDFTRDVLLSNFASIA

bcmD M223I LQIRPLTDYLAEPIFGRIATSFFEIASEAISKLNVAAIQDADLIVDLTMR**I**LLDNYQSIE

bcmD RAPPTNMILAQFDCFSRPFTRETIRALDAASLKELGTIIVATYDTTALSLLWTLTYLEDN

bcmD L130M TAPSSNMIKGQFTQYGYAFSPEAIGRLSRSQLKELGTIIVATYDTTALSLLWSMAYIETT

bcmD M223I AAPQGNLIRSQFGNFGIPFSRQGIATLTDAQLKELGTLIVATYDTTALSLLWTIAYLETT

bcmD PAEKERLLGVVDNPEQALDEAHLLVLEAIRLGGSNPTALWRRTNRPIRIRHRGTEVTIPA

bcmD L130M PRQRDRLIAALAGKEDASHVASALALEAVRLGGSNPTALWRQTKHPVTIRHSGIVVELPA

bcmD M223I PGARDRLLDSLGDHEVAQQTATMMVLEAIRLGGSNPTSLWRRSRRPVTILHRGSSVDIPA

bcmD NTMLWLDRRRANRDASLFPHAERFDTDNIRQLIRNQTSHGQAVSLLARNRYEINSFNMVN

bcmD L130M GTMIWLDRRHANRDAHSFPCPHAFDARNIEQITRNAVGN--TTSTLARNRYEINSFSMIN

bcmD M223I GTMFWLDRRLANRDPQRFARPNVFDPGNIEGIAN--HGGIEAASLLARNRYEINSFSMIN

bcmD THRSPRKCPGRLFSVREQALILTELYRLYKVCVTEADSTLAPHSSMPRPRRSGNIILTAR

bcmD L130M ADRNPRKCPGRLFAVRLQALALIEIYRRYDVVTSDIDTSLAKHSAMPRPRRPGTIILHAK

bcmD M223I TVRNPRKCPGRLFSVRAQALMLTELYRSNRVVVSGVSTGLMPGSAMPRPACPGSIRVEPI

bcmD AAAL

bcmD L130M AG--

bcmD M223I Q---

**Organisms associated with observed sequence variations**

*bcmE*: *Pseudomonas aeruginosa* N17-1

*bcmE* G39D: *Tistrella* sp.

*bcmE* L120I: *Burkholderia plantarii*

1 10 20 30 40 50 60

| | | | | | |

bcmE -----MSNILPSARMLDGMLHFKEEDGFSRARKLGAFHLEHPR**G**WDFSAGIALAQSYYLE

bcmE G39D MTAPHPTDILPTASLTAEGLCFDAPDGFDLARRRGAFHLAHPR**D**MDFTAGIRLARSYYLD

bcmE L120I -MNVSDTYEWAAARFDNGELIFDTSDGLTRALADGFFFVQKPAGFDLTPGDCFATNFYLD

bcmE PEVDADNAFRGFHRKDLG--KSLLGYSRTGADQDELLQIECGLWREYLPAPAADLLWAMN

bcmE G39D PDGGSGDAWRGFRDRDLS--PTLLGYSCTGADQDELLQIESHLWHRHLPAAAAALLWRMS

bcmE L120I GRGDANDTYRGFRAWNAERLAEREGYYCRDADQVEQFFLEGRFWNEVFPEALSRQAREMR

bcmE DLNRSV**L**SQLFALVGIEERHFDLIAG-----GMSSNQALQYCIFNHFRADSAHPVGLTAH

bcmE G39D GISRSVLRGLFAMAGVPATDVERVTGGLDPAGSSADGALQYCIFNHFRAEIDQPVGLTAH

bcmE L120I EFSVAV**I**RAILDRLDLPREILEKATG-----GILSGNGTYHLTFNHFRPQIRARG-LNTH

bcmE KDSGFTTLLYTTEPGLESLENGSWIPFDPMPGHFTLVLGHSFELLTDKSATPVQASYHRV

bcmE G39D KDSGFVTLLYTVEKGLESQDGAGWVPFDPLPGYFTLVLGHSLEVLTARLARPITASYHRV

bcmE L120I KDSGWITMLRSLEPGLEVLREREWVPLIPRPDAFVMNFGCAIEILTRDTRTPVAAVAHRV

bcmE RRMEPQER-KADRFTFGSYIGPRWDQDLYQISG-ETVKPVQSFLEFQKRKAAEMGYEFHP

bcmE G39D RTTRPRPAGMPDRFTFGTYIGPRWDQPLYEYTEDGRLTSSMSFLDFQKRKAAEMAYDFHP

bcmE L120I VEQQARPDGIPDRFSYALFVDSSLNPAQCEGLYRLDPQAGLVLAADFNTFLDEILANTYD

bcmE KVETAHR

bcmE G39D RVAEALG

bcmE L120I RDTEGLY

**Organisms associated with observed sequence variations**

*bcmF*: *Pseudomonas aeruginosa* N17-1

*bcmF* M71T: *Streptomyces sapporonensis*

*bcmF* R259H: *Actinokineospora spheciospongiae*

1 10 20 30 40 50 60

| | | | | | |

bcmF VNTKPKTASSPTLRAPVNLPAMPLESQV-HQYPPIEMARSRLLESRLVFDHPDGFEQARR

bcmF M71T ----MASPDSATLREPVVLPPMPGEHEARAAYPPIGLERSRVTGGRLVFDRDEGFDRALA

bcmF R259H ------MNQSMTYRDRVPLPPMPDEYEAGLRYPPANLERAQVRAGELVFDREDGLQRACA

bcmF HGFFLLEVPAR**M**DFCPGDLFVRNCFLPRAE-GALSTYTGFKECQIPGAYQGYFDREHDQW

bcmF M71T QGFFLVRIPEG**T**DPAAGDRFAAHFHEERAGGDPLDAYRGYRHVRVPGDYQGYFDREHDQW

bcmF R259H QGFFLVGIPEGVNTDPGDTLAAEFHRDRTG-DHLDPFRGWRDVEIPGDYQGYFDREHDQW

bcmF ENVYIERGNWSLIPEAVAALGVQMAELGIGVLRAVLAELDIPRAEWARLTSGLSEGQGHQ

bcmF M71T ENFYVERDNWDVLPSEVARVGRGMAGLGVTILRGVLEHLRLPREHWARVTGGLTEDRGHQ

bcmF R259H ENLYVESANWHLLPPGVAPLGHAMTDLGLLVLCSVLRGVGIPRDAWATVTSALSEKRGHQ

bcmF MFGFNHFRASKPIRGSKFHRDSGWVTVLRSTEPGLIAYIDGQLRSINPLPGHLIINFGSS

bcmF M71T MLAFNHFRSHKGVRGSKFHRDSGWVTVLRSVDPGLLALVDGRLWAVDPEPGHFIVNFGSS

bcmF R259H MLAFNHFRSTKDTRGSKFHRDSGWVTVLRSTEPGLLALIDGELRSVDPVPGHFIVNFGSS

bcmF MEVLSEHLSRKVHANVHGVA**R**TERAS-PDERYSYVVFLDSDLGGDIYRYGPAGAQKVQTV

bcmF M71T LEVLTERLDRPVRANVHGVVSTERAPGQPDRTSYVTFLDSDLTGTVYRFENGTPRPLQSV

bcmF R259H LEVLTERLPAPVRANVHGVA**H**TRRAPGTPDRTSYAIFLDSALGGTIHRWEDNTAHPVQSV

bcmF LEFAEQEVSRTYNDDILL

bcmF M71T AEFAGQEVGRTYDDSGAL

bcmF R259H AEFAEQEVNRTYDDTTHL

**Organisms associated with observed sequence variations**

*bcmG*: *Pseudomonas aeruginosa* N17-1

*bcmG* P109S: *Photorhabdus temperate*

*bcmG* R116Q: *Providencia heimbachae*

*bcmG* E123K: *Streptomyces platensis*

1 10 20 30 40 50 60

| | | | | | |

bcmG ----------------------------MNPNATYALASAELIDGKLRFDSSD-GFARAI

bcmG P109S ----------------------------MNINNRYEWASSKLQNGRLLFDTTD-GFRRAV

bcmG R116Q ----------------------------MNGNILNNWATGKLIKNDIIFDSPD-GIKKAF

bcmG E123K MSAARSADVPEIRRGRIYQDVYRKRVDTQLVTSTAALERARIEGDSLIFEGDDQAWKRAL

bcmG ADGFFFVKSP-SLDLAAGDTFARNFYLPRREGLGAPYQGFSQWTEDRLARREGYFSRDVD

bcmG P109S KDGFFFIKAPDTLNLSSGDLFATNFYLPKTGNSYDAYRDFKSWTTEKLAEREGYFLRNAD

bcmG R116Q QDGFFFIEKPHSLSLEAGDIFASEFYHSKMHKPNDEYRGFKTWTSEKLGKRQGYFIRSND

bcmG E123K GDGLFLLAIPPELDVRVGDAFSAQFYQG---ASAPPYGRYRELTSEHFGDALLGFHQRTN

bcmG QVEQFFLESRFWQTVFPG**P**LLRQAE**R**MRSFSL**E**VLRAVLAELDLPVELWDEATGRCLSMQ

bcmG P109S QVEQFFLEHRFWKNVFPL**S**LVTQAT**Q**MKEFGLEVLRAILSQLDIPAKLWNKATGGCLSGL

bcmG R116Q QVEQFFLEKAHWENVYPPQLIVQAN**Q**MQQFGIQIVNTIFSYLNIPSELWDKASGYSLSGK

bcmG E123K QIEQFLLERRFWDRYYPPEIAELGEGLTYLSR**K**VIRSVLCYAGIPEKDWEQATGGCTEAA

bcmG GTYHLTFNHFRSHVRARGLNVHKDSGWVTILRSLEPGLEVLREGDWLPVSPRPGEFIVNF

bcmG P109S GTYHLTFNHFRPTVRSRGLNIHKDSGWVTILRSLEPGLEILRKGEWLPIIPRPDTFIVNF

bcmG R116Q GSYHLTFNHYRPTIKARGLNTHKDSGWVTVLRSFEPGLEVLINQEWLPVIPKQNHFIVNF

bcmG E123K GSYHLTFNHYRPAHDATGLNSHKDDGFITLLRATAPGLEVSRADRWEAVVPEPGYFIINF

bcmG GCAMEILTRHSATPVAAVAHRVQEQLPG---QADRFSYALFVDSSLDPRTCPGLFRYLPG

bcmG P109S GCAMEILTRYATIPVAAVAHRVVEQKPGEQSGVDRFSYALFIDSSLDKNVSKGLYSYKHD

bcmG R116Q GCAMEILTKHAEYPVSAVVHRVSEQNENR--IKDRFSYALFVDSSLNKQVSDGLYRYSRQ

bcmG E123K GLSMEILTAHCAAPVRAIMHRVTRQTTDR------STFGHFSSSNCAPGSDEGIYRYLPG

bcmG HGLVLEADFEMFLNEILHNTYQENTQGLY---

bcmG P109S KGLVLETSFEEFLNKILHNTYEQHTQGLY---

bcmG R116Q QGLQLETDFEEFLNHIDHSTYEKGSSEFN---

bcmG E123K IGLDRVCGSRELIDENDSEIYEGTVNPEGRTP
