# Supplementary material for: Continuous bioactivity-dependent evolution of an antibiotic biosynthetic pathway
Source: Nat Commun. 2020 Aug 21;11:4202. doi: 10.1038/s41467-020-18018-2 (PMC7443133; doi:10.1038/s41467-020-18018-2)
Supplement: Supplementary file 2 — Peer Review File [file 41467_2020_18018_MOESM2_ESM.pdf]

Editorial Note: This manuscript has been previously reviewed at another journal that is not operating a transparent peer review scheme. This document only contains reviewer comments and rebuttal letters for versions considered at *Nature Communications.*
